# METTL3-IGF2BP3 as a glucose sensor in hyperglycemic microenvironment promotes tumorigenesis and glycolysis

**DOI:** 10.7150/ijbs.125135

**Published:** 2026-04-16

**Authors:** Long Liu, Yu Zhang, Zhiwei Shao, Xiaohong Zhao, Yuxi Huang, Qi Wang, Xingyu Liu, Xiang Zheng, Bo Zhou, Fabiao Zhang, Zhenzhen Gao, Sheng Yan

**Affiliations:** 1 Department of Hepatobiliary and Pancreatic Surgery, The Second Affiliated Hospital of Zhejiang University School of Medicine, Hangzhou, Zhejiang, 310000, China.; 2 Department of Surgery, Perelman School of Medicine, University of Pennsylvania, Philadelphia, Pennsylvania, 19104, USA.; 3 Department of Hepatobiliary and Pancreatic Surgery, Taizhou Hospital, Zhejiang University School of Medicine, Taizhou, Zhejiang, 317000, China.; 4 Department of Oncology, The First Affiliated Hospital of Anhui Medical University, Hefei, Anhui, 230001, China.; 5 Department of Oncology, The First Hospital of the University of Science and Technology of China, Hefei, Anhui, 230001, China.; 6 School of Pharmacy, Hangzhou Normal University, Hangzhou, Zhejiang, 310000, China.; 7 Department of Hepatobiliary and Pancreatic Surgery, Taizhou Hospital of Wenzhou Medical University, Taizhou, Zhejiang, 317000, China.

**Keywords:** Hepatocellular carcinoma, Glucose, Methylation, METTL3, IGF2BP3

## Abstract

Glycemic disorders, especially diabetes characterized by elevated blood glucose, significantly increase the risk of various cancers, including increasing the risk of hepatocellular carcinoma (HCC) by 239%. Diabetes mellitus has now been confirmed as an independent risk factor for the development of HCC, with T2DM increasing the risk by 4.59 times. However, little is known about mechanisms regulate glucose signal transduction in HCC. We investigated the relationship between high glucose levels and methylation-related gene expression in HCC. The effects of METTL3-IGF2BP3 on transcriptome profiles were used to identify downstream molecules. Proliferation, glucose uptake, lactate production, ATP levels, extracellular acidification rate, and oxygen consumption rate were examined in HCC cells. The mechanism by which METTL3-IGF2BP3 activates the PI3K-AKT pathway was explored. The results showed that m^6^A modification levels were significantly elevated in HCC cells under a hyperglycemic microenvironment, primarily due to transcriptional activation of c-MYC-initiated METTL3. Additionally, glucose could directly bind to IGF2BP3 and promote its function in recognizing m^6^A sites. SLC39A10 as a downstream molecule of METTL3-IGF2BP3, promoting the uptake of Zn^2+^ in HCC. METTL3, IGF2BP3, or SLC39A10 silencing inhibited proliferation, colony formation, glycolysis in HCC cells. Mechanistically, silencing METTL3-IGF2BP3 significantly reduced the methylation level and function of SLC39A10, decreased intracellular Zn²⁺ levels, inhibited ADAM17 activity, and thus attenuated EGFR phosphorylation-induced PI3K-AKT pathway activation. Concurrently, alterations in intracellular Zn²⁺ levels are associated with altered immune cell infiltration in the HCC tumor microenvironment. In conclusion, the METTL3-IGF2BP3 axis promotes HCC tumorigenesis by enhancing glycolytic reprogramming and remodeling the immunosuppressive tumor microenvironment.

## Introduction

Hepatocellular carcinoma (HCC) remains a leading cause of cancer-related deaths globally 1. While the incidence of HCC due to chronic viral hepatitis (HBV and HCV) is declining, cases arising from metabolic factors, such as obesity, metabolic syndrome, and type 2 diabetes mellitus (T2DM), are on the rise 2. Dysglycemia, especially diabetes characterized by elevated blood glucose, significantly increases the risk of various cancers, including a 239% heightened risk of HCC 3. Recent studies have established diabetes as an independent risk factor for HCC development, with type 2 diabetes increasing the risk by 4.59 times 3, 4; notably, the longer the duration of diabetes, the higher the HCC risk (2.96, 6.08, and 7.52 times for durations of 0-2 years, 2-10 years, and more than 10 years, respectively). Current research on the link between diabetes mellitus and HCC development primarily focuses on insulin resistance, glucose and lipid metabolism disorders, and abnormal inflammatory mediator release 5. However, the direct role of high extracellular glucose as a signaling molecule in regulating HCC progression and its underlying signaling mechanisms remain to be fully elucidated.

RNA methylation modifications account for more than 60% of all RNA modifications, with N6-methyladenosine (m⁶A) being the most prevalent 6. Numerous studies have demonstrated the critical role of m⁶A modification in tumorigenesis, progression, metastasis, and recurrence 7; as a primary energy source for tumor cells, glycolysis is also regulated by m⁶A 8. For instance, in breast cancer, the m⁶A “writer” METTL3 influences tumorigenesis and glycolysis by mediating m⁶A methylation of the tumor suppressor LATS1 9; the m⁶A “eraser” ALKBH5 promotes glycolysis and resistance to anti-HER2 therapy in breast cancer by demethylating m⁶A on GLUT4 mRNA 10; additionally, the m⁶A status of PDK4 can affect glycolysis in both breast and liver cancer cells 11. Nevertheless, these studies all focus on the regulation of key glycolytic molecules by m⁶A “writers” or “erasers”, and overlook the role of m⁶A “readers” in glucose signal sensing—specifically, whether glucose can directly bind to m⁶A readers to modulate their ability to recognize m⁶A modification sites, thereby mediating the intracellular transmission of glucose signals. This represents a significant research gap.

Previous studies have shown that glucose, in addition to its role as an energy substrate, can act as a signaling molecule by directly binding to proteins (especially RNA-binding proteins) to alter their structure and function 12. For example, during epidermal differentiation, glucose binds to the ATP-binding domain of DDX21, altering its conformation, inhibiting helicase activity, and disrupting dimerization; elevated glucose levels also induce the relocation of DDX21 from the nucleolus to the nucleoplasm 13. Moreover, glucose binding to NSUN2 promotes m⁵C RNA methylation by TREX2, thereby limiting cytoplasmic dsDNA accumulation and cGAS/STING activation, ultimately promoting tumorigenesis and resistance to anti-PD-L1 immunotherapy 12. However, the RNA-binding proteins bound by glucose in these studies do not belong to the “readers” in the m⁶A modification regulatory network. In-depth research on “glucose directly binding to m⁶A readers in a high-glucose environment to regulate m⁶A modification function and promote HCC progression” is still lacking.

Despite the established link between hyperglycemia and hepatocellular carcinoma progression, the epitranscriptomic mechanism coupling extracellular glucose sensing to intracellular oncogenic reprogramming in HCC remains largely uncharacterized. We hypothesize that the METTL3-IGF2BP3 m⁶A regulatory axis functions as a glucose sensor in HCC, driving hyperglycemia-mediated tumor progression via modulating zinc homeostasis. This study aims to systematically clarify the functional role and molecular mechanism of the METTL3-IGF2BP3-SLC39A10 axis in hyperglycemia-related HCC, and to provide potential therapeutic targets for HCC patients with hyperglycemia.

## Materials and Methods

### Patients

Eighty matched paraffin-embedded samples of normal tissue and HCC, as well as sixteen fresh tissue samples, were collected from the Second Affiliated Hospital of Zhejiang University (Table S1). Written informed consent was obtained from all patients, and the study adhered to the guidelines of the Institutional Review Board (IRB) of the Second Affiliated Hospital of Zhejiang University (Ethics No: 2024-0397).

### Experiment details

Human HCC cell lines Hep3B and Huh7, along with human embryonic kidney cells 293T, were obtained from the Shanghai Institutes for Life Sciences Cell Bank, Chinese Academy of Sciences. Reverse transcription and real-time PCR were conducted using PrimeScript reagents (Takara Biotechnology Co., Ltd., Dalian, China). Supplementary Table S2 and S3 list the primer sequences employed. For Western blot analysis, tissue or cell samples were lysed with RIPA buffer and quantified using the BCA method. Proteins were separated by SDS-PAGE electrophoresis and subsequently transferred to a PVDF membrane. The membrane was blocked with 5% skimmed milk and then incubated with primary antibody overnight. Detailed antibody information is provided in the Supplementary Material (Table S4). The Supplementary Information outlines the procedures for cell colony formation assays, Cell Counting Kit-8 (CCK-8) assays, Seahorse metabolic assays, immunofluorescence, ELISA, and RNA stability analysis.

The biotin-glucose pull-down assay was performed with the core operating system for biotinylated glucose-protein binding established in reference 12, and the standardized workflow, quality control system and competitive control setting for biotinylated metabolite pull-down described in reference 14. Magic Dynabeads MyOne Streptavidin T1 (Thermo Fisher) were incubated with free biotin or biotin-labeled glucose for 30 minutes at room temperature, followed by overnight incubation with cell lysates at 4 degrees Celsius. Subsequently, the cells were analyzed by immunoblotting. Chromatin immunoprecipitation (ChIP) assays were performed using the Magna ChIP Kit (Millipore, Bedford, MA, USA). Hep3B and Huh-7 cells were treated with formaldehyde to crosslink DNA and proteins. Cell lysates were then sonicated to generate 200-300 bp chromatin fragments, and immunoprecipitation was carried out using c-MYC antibody or IgG as a control. The precipitated chromatin DNA was recovered and analyzed by quantitative PCR (qPCR). m^6^A immunoprecipitation was performed following a previously established method 15. For the luciferase reporter assay, 200 ng of the signal luciferase reporter gene (OriGene), 5 ng of the pRL-TK kidney reporter plasmid, and siRNA (siNC or siSLC39A10) or pGL-SLC39A10 (wt, mut1, or mut2) plasmid were transfected. Dual luciferase reporter gene assays were conducted 48 hours post-transfection using the Dual-Luciferase Reporter Gene Assay Kit (E1910, Promega, USA).

All animal experiments were approved by the Animal Research Committee of the Ethics Committee of the Second Affiliated Hospital of Zhejiang University. Diabetes was induced in 4-week-old BALB/c nude mice using streptozotocin (STZ) at a concentration of 100 mg/kg on Day 0 and Day 7. Blood samples were taken from the tail vein and measured with NB-loT (Hangzhou, China). The mice with blood glucose > 300 mg/dL were recognized as successful in diabetic modeling. And then approximately 2×10⁷ Huh7 cells, stably transfected with the specified interventions for each subgroup, were subcutaneously injected into the hind abdomen of mice. Tumors were measured every 5 days for 6 weeks using digital calipers, with volume calculated as (length×width²) / 2. After 6 weeks, mice were euthanized, tumors were weighed, and excised tumor tissue was subjected to H&E analysis.

### Statistical analysis

Statistical analyses were conducted using SPSS version 24.0 (SPSS Inc., Chicago, IL, USA) or GraphPad Prism 10.0 software (GraphPad, CA, USA). All *in vitro* experiments were performed independently with at least biological replicates. Data were presented as mean ± SD. Statistical tests employed included Student's t-test, one-way ANOVA, two-way ANOVA, χ² test, Spearman's correlation analysis, and others. *p*-value less than 0.05 was statistically significant.

## Results

### High glucose upregulates m^6^A modification in HCC via c-MYC-mediated transcriptional activation of METTL3

**Figure 1:**
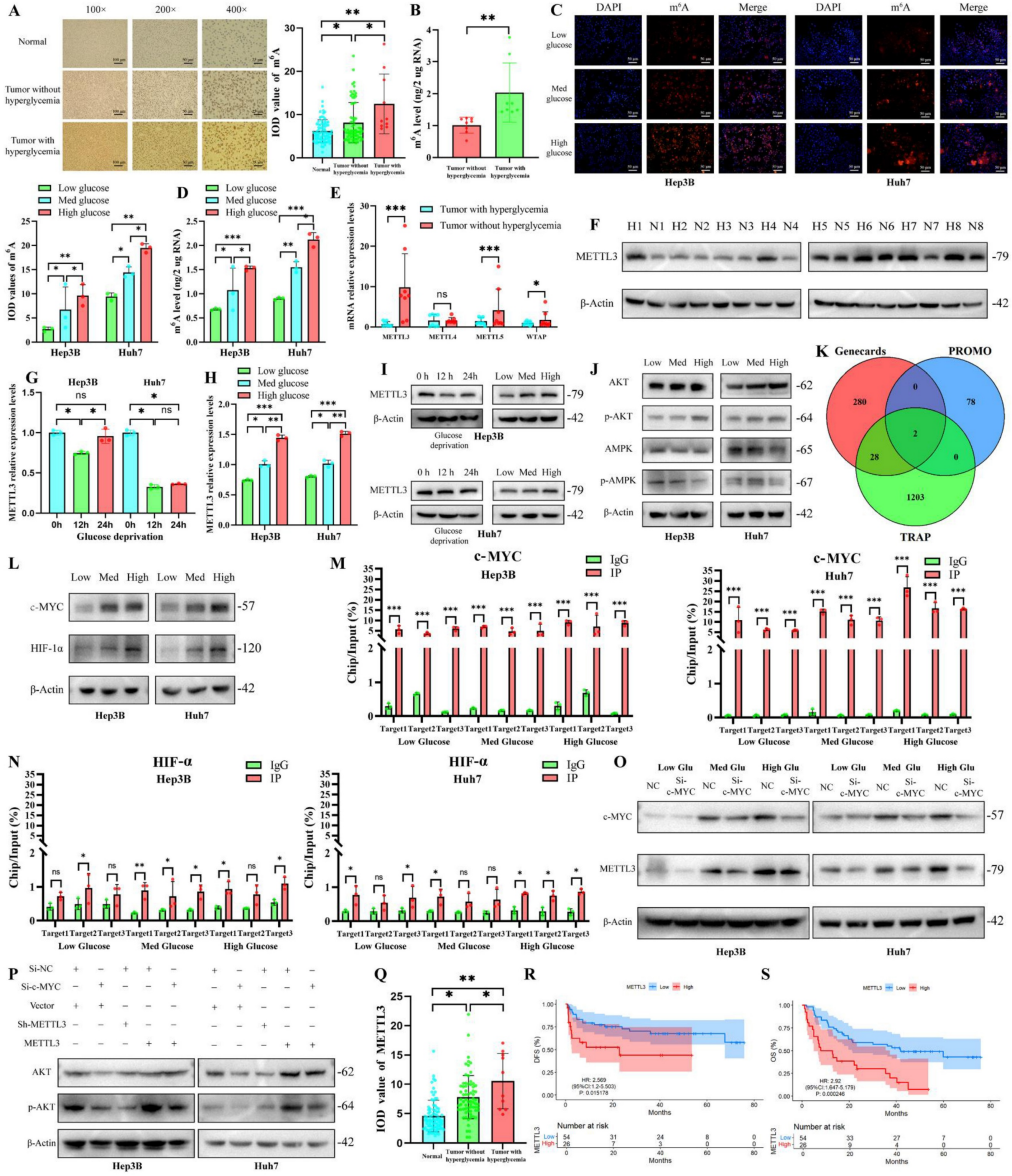
** High glucose levels promoted the expression of METTL3 in HCC.** Immunohistochemical staining of m^6^A in 80 pairs of HCC patients (A) and ELISA of m^6^A in 16 patients (B) suggest that hyperglycemia promoted m^6^A levels in HCC patients; The 100×, 200×, and 400× scale bars were 100 μm, 50 μm, and 25 μm, respectively; Immunofluorescence (C) and ELISA (D) of m^6^A of HCC cells at different glucose concentrations, Scale bar=50 μm; qPCR (E) and western blot (F) demonstrated hyperglycemia promoted METTL3 expression in HCC patients; METTL3 expression was affected by glucose deprivation time (G) and glucose concentration (H); (J) Glucose concentration affected AKT/AMPK expression and phosphorylation in HCC cells; (K-L) c-MYC and HIF-1α represent METTL3 transcription factors and exhibited changes with different glucose concentrations; c-MYC regulated METTL3 transcription (M), whereas HIF-1α did not (N); (O) METTL3 expression was affected by glucose concentration and c-MYC; (P) A regulatory relationship was observed between c-MYC and METTL3 in the activation of the AKT pathway; (Q-S) METTL3 expression was significantly associated with hyperglycemia and prognosis in HCC patients. *p < 0.05. **p < 0.01. ***p < 0.001. NS, no significance.

To investigate the effects of a high-glucose environment on m^6^A modification in HCC, we compared m^6^A levels in HCC tissues from patients with and without hyperglycemia. Results revealed significantly elevated m^6^A modification in HCC tissues from hyperglycemic patients (Fig. 1A, B). Subsequently, we examined the m^6^A expression levels in six common HCC cell lines under high-glucose conditions. The results demonstrated that Hep3B and Huh7 cells exhibited significantly upregulated m^6^A expression under high-glucose stress, and thus were selected for subsequent experiments (Figures S1A). Similarly, compared with the low-glucose (5 mmol/L)/medium-glucose (15 mmol/L) environment, HCC cells cultured in the high-glucose (25 mmol/L) environment exhibited increased m^6^A modification (Figures 1C, D). Given that m^6^A modification is mediated by “writers” in the regulatory network 16, we examined the expression of m^6^A-modifying “writers” genes. *METTL3* was identified as the most significantly upregulated gene in high glucose conditions (Fig. 1E, F) (All Western blot quantifications in this study are provided in Figure S6). We further explored the effects of high glucose on METTL3 expression at the cellular level. Glucose deprivation experiments demonstrated a peak decline in METTL3 expression after 12 hours of low glucose incubation (Fig. 1G). Additionally, METTL3 expression increased with rising glucose concentrations in the culture medium (Fig. 1H, I). Hyperglycemic microenvironment is known to primarily regulate glucose metabolism in HCC 17. We assessed changes in glucose metabolism-related signaling pathways and transcription factors in HCC cells cultured in high glucose 18. Results indicated activation of the PI3K-AKT pathway and inhibition of AMPK (Fig. 1J). Transcription factor database analyses (Genecards, PROMO, and TRAP) suggested that HIF-1α and c-MYC might regulate METTL3 transcription (Fig. 1K). Indeed, both HIF-1α and c-MYC were upregulated in high glucose conditions (Fig. 1L).

To determine the role of HIF-1α and c-MYC in METTL3 transcription, we performed chromatin immunoprecipitation-quantitative PCR (ChIP-qPCR) analysis. Only c-MYC was found to be involved in METTL3 transcription in a hyperglycemic microenvironment (Fig. 1M, N). Meanwhile, the specificity of the c-MYC antibody was validated (Fig. S1B). Knockdown of c-MYC in HCC cells decreased METTL3 expression, with a more pronounced inhibitory effect at higher glucose concentrations (Fig. 1O, S1C). Luciferase reporter assays further confirmed the direct regulation of METTL3 transcription by c-MYC (Fig. S1D). Furthermore, c-MYC and METTL3 were shown to have a regulatory relationship in the activation of the AKT pathway (Fig. 1P). Finally, we analyzed the differential expression and clinical significance of METTL3 in HCC patients. METTL3 expression was lowest in paracancerous tissues and highest in patients with hyperglycemia (Fig. 1Q). High METTL3 expression was associated with poorer overall survival (OS) and disease-free survival (DFS) in HCC patients (Fig. 1R-S, S1E). Taken together, these findings establish a strong link between the hyperglycemic microenvironment and m^6^A modification, identifying METTL3 as a key mediator of m^6^A modification in HCC under high glucose conditions.

### Glucose directly binds to the KH3-4 domain of IGF2BP3 to enhance its m^6^A reader function

**Figure 2:**
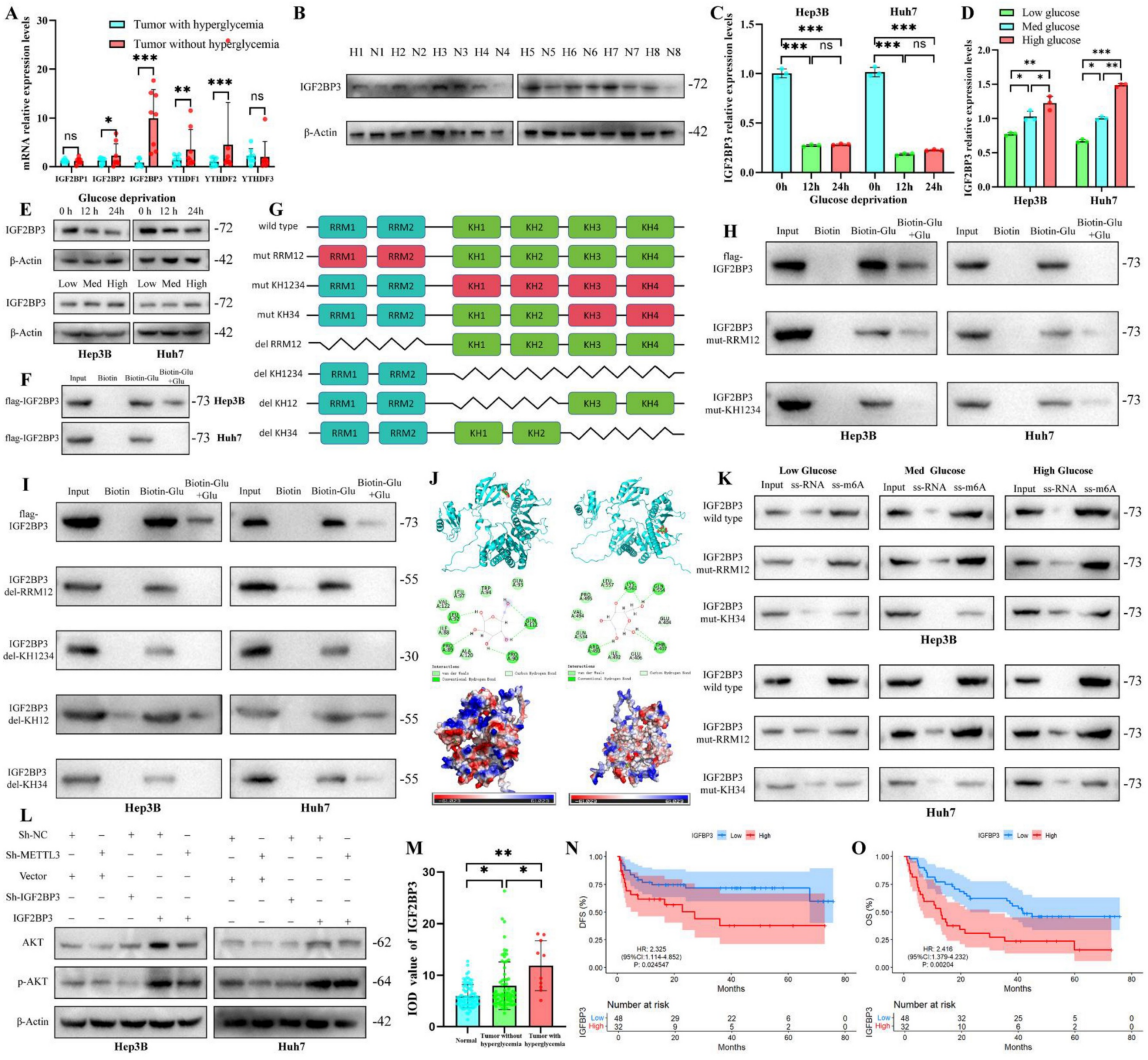
** Glucose binds directly to the KH34 domain of IGF2BP3, enhancing its m^6^A recognition function.** PCR (A) and western blot (B) of 16 HCC patients detected IGF2BP3 expression in HCC samples; Assessing RNA(C-D) and protein(E) level of IGF2BP3 in HCC cells in different glucose deprivation time and glucose concentration; (G) Schematic diagram of the mutations in the structural domain of the IGF2BP3 protein; (H-I) Biotin-labeled glucose binding to IGF2BP3 protein with RRM1-2(H), KH-1-2-3-4(H), KH-1-2(I) or KH-3-4(I) structural domain mutations assessed by pull down and Western blot assay; Glide module in Schrödinger Maestro predicts glucose and IGF2BP3 protein binding by molecular docking predictive graphs(J); Pull down and Western blot assay to evaluate the binding ability of IGF2BP3 proteins with different structural domain deletions and biotin-m^6^A probes(K); Western blot analysis of the effects of METTL3 and IGF2BP3 on the PI3K/AKT pathway(L); (M-O) IGF2BP3 protein level and its prognostic value. *p < 0.05. **p < 0.01. ***p < 0.001. NS, no significance.

In the m^6^A methylation regulatory system, the “writer” enzyme catalyzes the addition of the m^6^A modification to RNA, and the “reader” proteins subsequently recognize and interpret this information 16, 19. We analyzed the expression of major m⁶A reader proteins in HCC tissues, and found that IGF2BP3 was significantly upregulated in hyperglycemic HCC samples (Fig. 2A, B), with its expression elevated in a glucose concentration-dependent manner in HCC cells (Fig. 2C-E). Given that glucose can directly bind to RNA-binding proteins to exert metabolism-independent functions 13, we hypothesized that glucose could directly bind to the m⁶A reader IGF2BP3 20 to modulate its structure and function.

To test this hypothesis, we performed biotin-glucose pull-down assays and confirmed the specific binding between IGF2BP3 and glucose in HCC cell lysates (Fig. 2F). Truncation mutation assays mapped the RRM1-2 and KH3-4 domains as the key regions responsible for glucose binding (Fig. 2G-I), which was further supported by molecular docking prediction and site-specific mutation validation (Fig. 2J, K). The direct physical interaction between IGF2BP3 and glucose was finally confirmed by isothermal titration calorimetry (ITC) assay, with a dissociation constant (KD) of 12.3 μmol/L (Fig. S2A). Functional validation showed that only mutation of the KH3-4 domain, but not the RRM1-2 domain, impaired the m⁶A recognition capacity of IGF2BP3 (Fig. 2K), and consistently reduced its binding to the downstream target SLC39A10 mRNA (Fig. S2B).

And then we observed a positively regulatory relationship was observed between IGF2BP3 and the activation of the PI3K/AKT pathway (Fig.2L, S2C). Additionally, we analyzed the relationship between IGF2BP3 and METTL3 and its clinical value. IGF2BP3 was found to be associated with METTL3 expression in HCC (Fig.S2D), Meanwhile, IGF2BP3 expression was lowest in normal tissue and highest in patients with hyperglycemia (Fig. 2M). Moreover, high IGF2BP3 expression was associated with poor OS and DFS in HCC patients (Fig. 2N, O, S2E). These findings highlight a novel role for glucose in regulating m^6^A modification, independent of glucose metabolism and through direct binding to IGF2BP3.

### The METTL3-IGF2BP3 axis drives HCC proliferation and glycolytic reprogramming under high-glucose conditions

**Figure 3:**
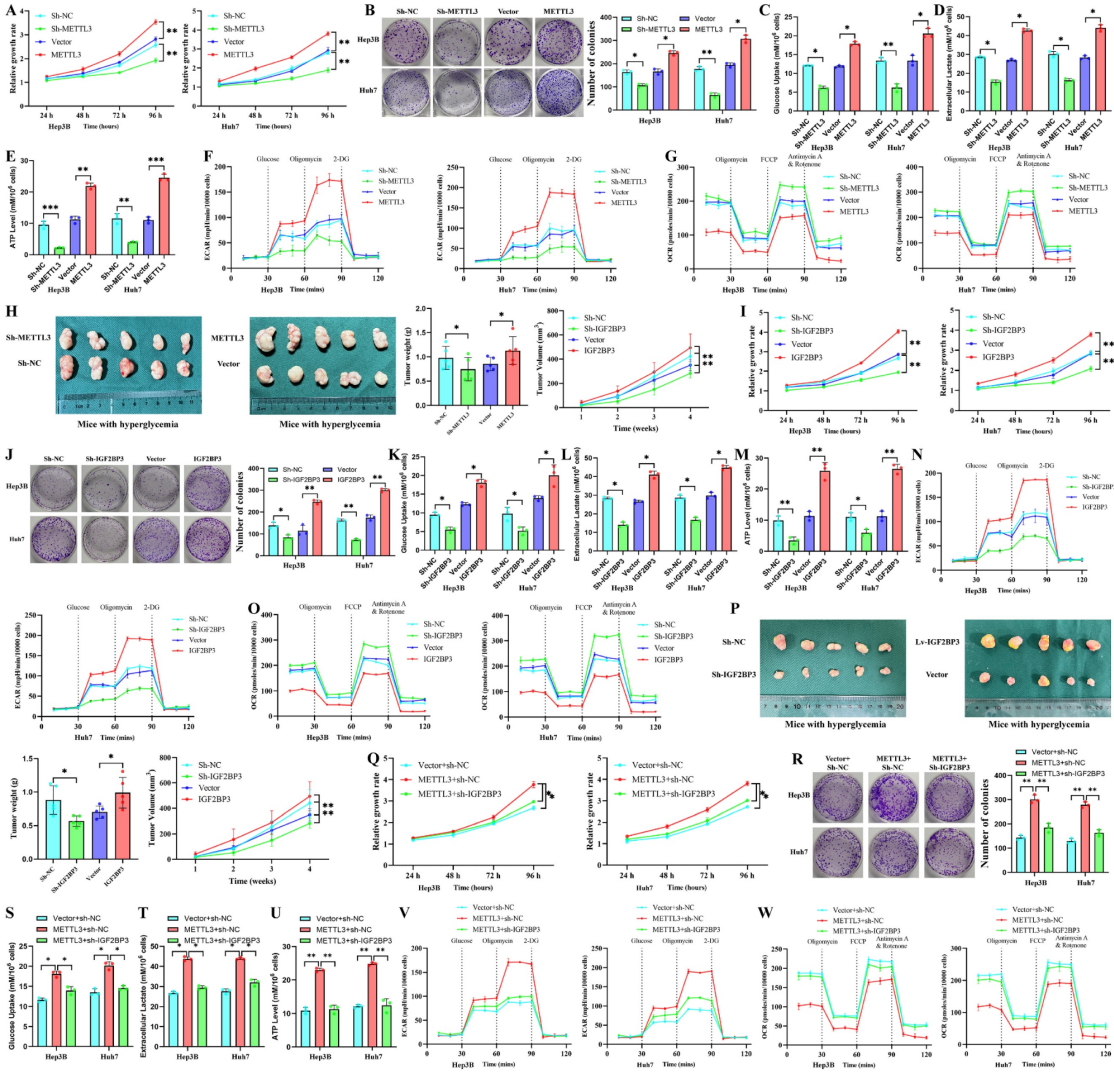
** METTL3 and IGF2BP3 affect the proliferation and glucose metabolism of HCC cells.** Effects on biological functions of HCC cells after intervention with METTL3 expression: proliferation (A) and clonogenic ability (B) of HCC cells, while glucose uptake (C), lactate accumulation (D), ATP production (E), and ECAR (F) were significantly decreased and OCR (G) was increased; (H) The effect of METTL3 on the proliferation rate and volume in xenograft tumors; Interventing IGF2BP3 assessed HCC cell proliferation (I), cloning (J), glucose uptake (K), lactate accumulation (L), ATP production (M), and ECAR (N), and OCR (O) in HCC cells as well as proliferation rate and volume of xenograft tumors *in vivo* (P); (Q-W) Knockdown of IGF2BP3 reverses the enhanced proliferation and glucose metabolism caused by METTL3 overexpression. Data are presented as the mean ± SD from three independent experiments. *p < 0.05. **p < 0.01. ***p < 0.001. NS, no significance.

To assess the effects of METTL3 and IGF2BP3 on HCC cell proliferation and glucose metabolism, we conducted *ex vivo* experiments. In our study, we observed a significant increase in HCC cell viability and clonogenicity when METTL3 expression was elevated. Conversely, inhibiting METTL3 expression significantly impaired cell viability and proliferation (Figure 3A, B). Furthermore, overexpression of METTL3 in HCC cells led to a notable increase in glucose uptake (Figure 3C), lactate accumulation (Figure 3D), ATP production (Figure 3E), and extracellular acidification rate (ECAR) (Figure 3F), while oxygen consumption rate (OCR) decreased (Figure 3G). This enhancement of glycolytic metabolism in tumor cells was substantially suppressed when METTL3 expression was reduced. *In vivo* experiments demonstrated that tumors derived from HCC cells with overexpressed METTL3 exhibited significantly enhanced proliferation and reduced necrosis (Figure S3A). Conversely, HCC cells with suppressed METTL3 formed smaller, lighter tumors (Figure 3H). Subsequently, we manipulated IGF2BP3 expression in HCC cells. Elevating IGF2BP3 levels resulted in significantly higher proliferative capacity (Figure 3I, J) and increased glucose consumption and extracellular acidification (Figure 3K-O). However, inhibiting IGF2BP3 expression reversed these effects on proliferation and metabolic activity in hepatocellular carcinoma cells in subcutaneous xenograft tumor experiments, IGF2BP3 overexpression led to larger, heavier tumors with reduced necrosis (Fig.3P, S3B). Furthermore, we found that METTL3 and IGF2BP3 are cross-regulated in glucose metabolism and proliferation. Knockdown of IGF2BP3 effectively reversed the hypermetabolic and proliferative state induced by METTL3 overexpression (Fig. 3Q-W). In addition, we investigated the effects of diabetes itself on HCC cell survival and tumorigenesis. The results demonstrated that diabetes could promote HCC tumorigenesis to a certain extent (Fig. S3C). These findings strongly support the pro-proliferative and pro-metabolic effects of METTL3 and IGF2BP3 on HCC cells. Targeting these molecules could potentially inhibit HCC cell viability.

### IGF2BP3 promotes SLC39A10 stabilization and expression in an m^6^A-dependent manner

**Figure 4:**
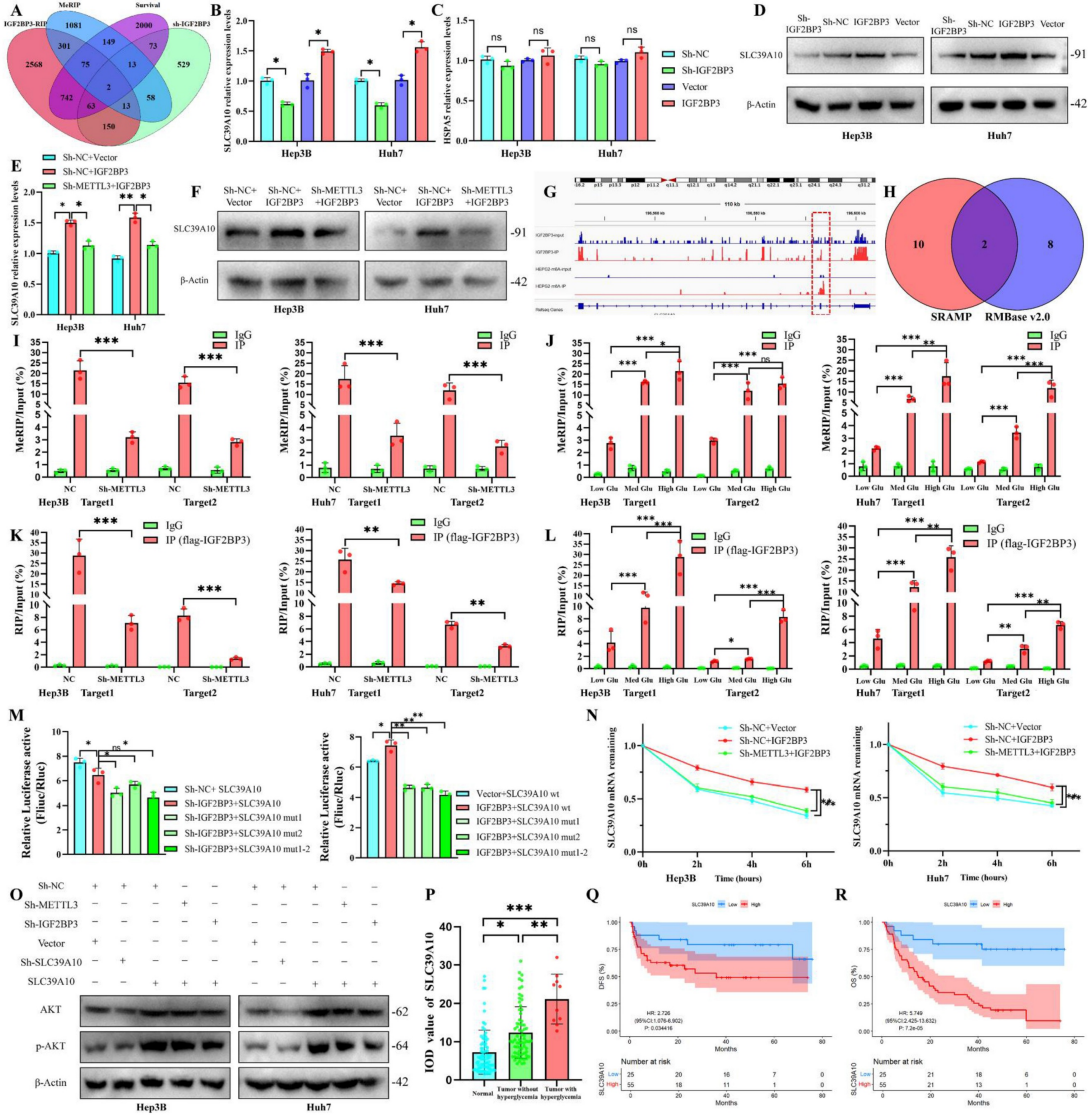
** IGF2BP3 promotes SLC39A10 stabilization and expression in an m^6^A-dependent manner.** (A) MeRIP-seq data from METTL3-silenced cells, IGF2BP3-RIP-seq data, Sh-IGF2BP3-RNA-seq data, and the overlap of HCC survival-associated genes; the effects of IGF2BP3 on SLC39A10 (B) and HSPA5 (C) using PCR and Western blot (D); Assessing the influence of METTL3-IGF2BP3 axis on SLC39A10 protein by qPCR (E) and Western blot (F); IGF2BP3-RIP-seq and MeRIP-seq datasets to identify m^6^A modification sites (G); SRAMP and RMBase predicted m^6^A sites of SLC39A10 (H); MeRIP-qPCR analyze the presence of m^6^A modifications and influenced by METTL3 expression (I) or glucose concentration (J); FLAG-IGF2BP3 RIP-qPCR detected SLC39A10 during both METTL3 knock down (K) and different glucose concentration (L); the interaction between IGF2BP3 and the two m^6^A modification sites in the SLC39A10 3'UTR, analyzed by dual-luciferase gene reporter assay (M); Actinomycin D intervention experiments revealed SLC39A10 RNA stability influenced by METTL3 and IGF2BP3 (N); SLC39A10, IGF2BP3, and METTL3 in activating the PI3K/AKT pathway detected by Western blot (O); Analyzed SLC39A10 expression (P) and prognosis (Q-R) in HCC patients samples. *p < 0.05. **p < 0.01. ***p < 0.001. NS, no significance.

To elucidate the downstream molecular mechanisms of METTL3-IGF2BP3, we analyzed MeRIP-seq data from METTL3-silenced cells, IGF2BP3-RIP-seq data, Sh-IGF2BP3-RNA-seq data, and the overlap of HCC survival-associated genes, ultimately identifying HSPA5 and SLC39A10 (Fig. 4A). We then examined the effects of IGF2BP3 on SLC39A10 and HSPA5 using PCR. While IGF2BP3 significantly regulated SLC39A10 (Fig. 4B), it had minimal impact on HSPA5(Fig. 4C). Further investigations were conducted to evaluate the effect of HSPA5 on glucose metabolism. The results showed that overexpression/knockdown of HSPA5 exerted no significant effect on ECAR, OCR, glucose uptake, lactate production, or ATP generation in HCC cells (Fig. S4A-C), thus HSPA5 was excluded. Moreover, SLC39A10 levels were consistently altered with IGF2BP3 at the protein level (Fig. 4D). Notably, knocking down METTL3 reversed the upregulation of SLC39A10 caused by IGF2BP3 overexpression (Fig. 4E, F), solidifying SLC39A10 as a downstream target of METTL3-IGF2BP3. We further analyzed IGF2BP3-RIP-seq and MeRIP-seq datasets to identify m^6^A modification sites. Our analysis revealed that IGF2BP3 binding sequences and m^6^A-modified regions were predominantly enriched in the 3'UTR of SLC39A10 RNA (Fig. 4G). Using SRAMP and RMBase, we predicted two high-confidence m^6^A sites in the 3'UTR region of SLC39A10 (Fig. 4H). We designed primers targeting the two identified m^6^A sites and performed MeRIP-qPCR analysis in HCC cells. Our results confirmed the presence of m^6^A modifications at these sites, which were reduced when METTL3 expression was inhibited or glucose concentration was decreased (Fig. 4I, J). Analyzed by anti-IGF2BP3-RIP-qPCR, we detected IGF2BP3 binding with SLC39A10 RNA. Moreover, this binding was regulated by both METTL3 (Fig. 4K) and glucose concentration (Fig. 4L). To validate the interaction between IGF2BP3 and the two m^6^A modification sites in the SLC39A10 3'UTR, we performed a dual-luciferase gene reporter assay. Mutating each m^6^A sites suppressed fluorescence signals, with the greatest suppression observed when both sites were mutated simultaneously (Fig. 4M). This further supports the interplay between IGF2BP3 and SLC39A10 in HCC cells. Actinomycin D intervention experiments revealed that overexpressing IGF2BP3 promoted SLC39A10 RNA stability, an effect reversed by knocking down METTL3 (Fig. 4N). We investigated the influence of SLC39A10, IGF2BP3, and METTL3 in activating the PI3K/AKT pathway and found that elevating SLC39A10 levels activated the PI3K-AKT pathway in HCC cells while knocking down METTL3 or IGF2BP3 inhibited PI3K-AKT signaling induced by SLC39A10 overexpression (Fig. 4O). SLC39A10 expression was lowest in paracancerous tissue and highest in patients with hyperglycemia (Fig. 4P). High SLC39A10 expression was associated with poor OS and DFS in HCC patients (Fig. 4Q, 4R, S4D). Besides, the expression of SLC39A10 was positively associated with IGF2BP3 and METTL3 (Fig. S4E). These findings demonstrate that IGF2BP3 could promote SLC39A10 stability and expression in an m^6^A-dependent manner, ultimately impacting the PI3K-AKT pathway.

### METTL3-IGF2BP3 promotes proliferation and glucose metabolism by regulating SLC39A10 in HCC cells

**Figure 5:**
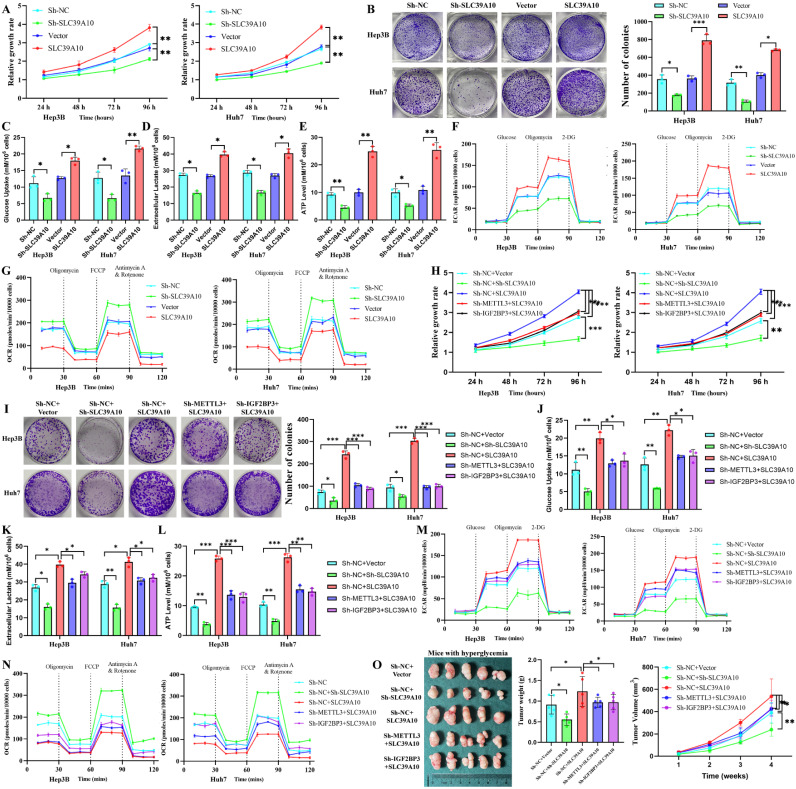
** METTL3/IGF2BP3 can affect the proliferation and glucose metabolism of HCC cells via SLC39A10.** Influencing the expression of SLC39A10 assessed tumor biological functions: proliferation (A), cloning (B), glucose uptake (C), lactate accumulation (D), ATP production (E), ECAR (F) and OCR (G); Knockdown of METTL3 or IGF2BP3 reversed proliferative activity (H/I), enhanced glucose metabolism (J-N), and tumor proliferation (O) induced by SLC39A10 overexpression. Data are presented as the mean ± SD from three independent experiments. *p < 0.05. **p < 0.01. ***p < 0.001. NS, no significance.

To investigate the effects of METTL3-IGF2BP3 regulation of SLC39A10 expression on HCC cell proliferation and glucose metabolism, we conducted *in vitro* experiments. Overexpressing SLC39A10 in HCC cells enhanced proliferation, glucose uptake, lactate accumulation, ATP production, ECAR, and reduced OCR (Fig. 5A-G). Conversely, knocking down METTL3 or IGF2BP3 effectively reversed the enhanced glucose metabolism and proliferation induced by SLC39A10 overexpression (Fig. 5H-N). During *in vivo* experiments, tumors derived from SLC39A10-overexpressing HCC cells were larger, heavier, and exhibited less necrosis (Fig. 5O, S3D). Knocking down METTL3 or IGF2BP3 reversed the proliferative activation of HCC caused by SLC39A10 overexpression (Fig. 5O). These findings collectively demonstrate that METTL3-IGF2BP3 can influence glucose metabolism and proliferation in HCC cells by regulating SLC39A10 expression.

### SLC39A10 affects ADAM17 function and the PI3K-AKT signaling pathway by regulating Zn^2+^ homeostasis in HCC cells

**Figure 6:**
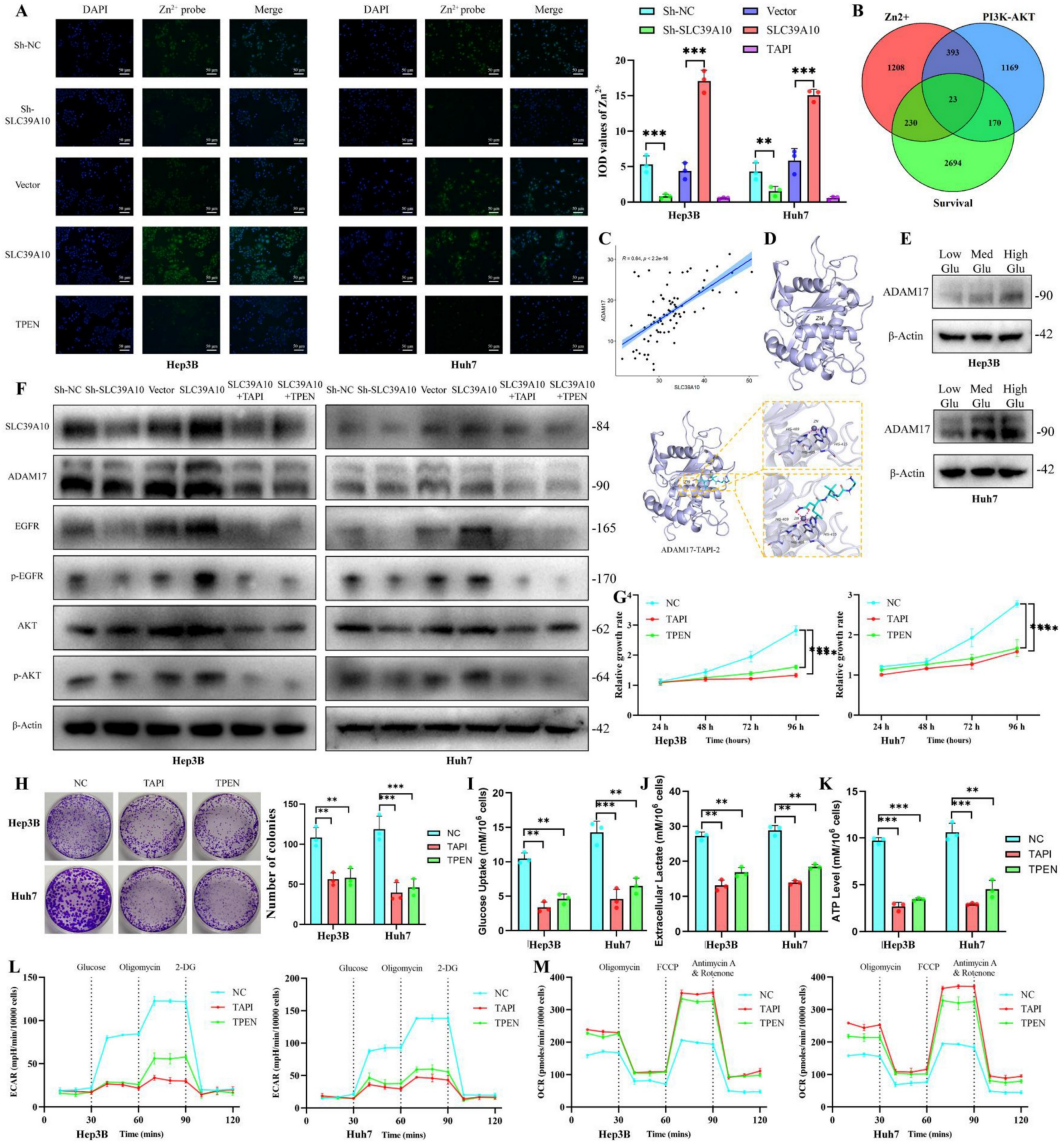
** SLC39A10 regulates ADAM17 and the PI3K-AKT signaling by affecting zinc ion homeostasis.** Analysed SLC39A10 effected on zinc ion uptake by Zn^2+^probe in HCC cells (A), Scale bar=50 μm; Zn ion-related proteins, PI3K/AKT-related proteins and overall survival genes overlapping (B); ADAM17 correlated with SLC39A10 expression analyzed by TCGA-LIHC dataset (C); Schematic diagram of the binding of ADAM17 protein and Zn ions (D); ADAM17 levels assessed in different glucose concentration (E); Western blot detected ADAM17 and phosphorylation of EGFR and AKT in SLC39A10 silence, chelating zinc ions, or ADAM17 inhibitors conditions (F), Chelation of zinc ions or the addition of ADAM17 inhibitor decreased the proliferation (G, H) and glucose metabolism of hepatocellular carcinoma cells (I-M). *p < 0.05. **p < 0.01. ***p < 0.001. NS, no significance.

SLC39A10 is a zinc ion transport protein that transports Zn ions from the extracellular environment into the cell 21, 22. In the present study, knockdown or overexpression of SLC39A10 significantly affected intracellular zinc ion levels (Fig. 6A). SLC39A10 acts as a downstream target gene of METTL3-IGF2BP3 for the activation of the PI3K/AKT pathway in high glucose culture (25 mmol/L). Based on this information, we identified Zn ion-related proteins and PI3K/AKT-related proteins from GeneCard, combined with genes associated with overall survival, to obtain overlapping genes (Fig. 6B). Among these overlapping genes, we found that ADAM17 could activate the PI3K/AKT signaling pathway by activating EGFR as previously reported 23, 24. ADAM17 has also been reported to play an important role in tumor maintenance and progression in HCC 25. Moreover, the expression of ADAM17 was highly consistent with that of SLC39A10 (Fig. 6C). ADAM17 is a metalloproteinase (Fig. 6D), and its enzymatic activity is dependent on the presence of Zn ions 26. We found that in HCC cells, ADAM17 levels increased with elevated glucose concentration in the culture environment (Fig. 6E). The ADAM17 protein level was suppressed upon either lowering the environmental zinc ion concentration using the Zn ion chelator N,N,N',N'-tetrakis (2-pyridinylmethyl)-1,2-ethanediamine (TPEN) (Fig. 6F) or competitively occupying the Zn ion binding site by the ADAM17 inhibitor tumor necrosis factor-α protease inhibitor-2 (TAPI-2) 27. We further explored the effect of the SLC39A10-ADAM17 axis, bridged by Zn ions, on the PI3K-AKT signaling pathway. We found that overexpression of SLC39A10 activated the EGFR-PI3K-AKT signaling pathway, but this activation could be reversed by chelating intracellular Zn²⁺ with TPEN or using the ADAM17 inhibitor TAPI-2 (Fig. 6F). Additionally, exogenous addition of zinc ions can reverse the inhibitory effect of SLC39A10 knockdown on the AKT pathway (Fig. S5A). Additionally, inhibiting Zn ions with TPEN or ADAM17 with TAPI reduced the proliferation and glucose metabolism of HCC cells (Fig. 6G-M). These results revealed that SLC39A10 could regulate ADAM17 and activate the PI3K-AKT pathway by regulating zinc ion homeostasis in HCC cells.

### The METTL3-IGF2BP3-SLC39A10 regulatory axis is associated with the tumor immune microenvironment in HCC

**Figure 7:**
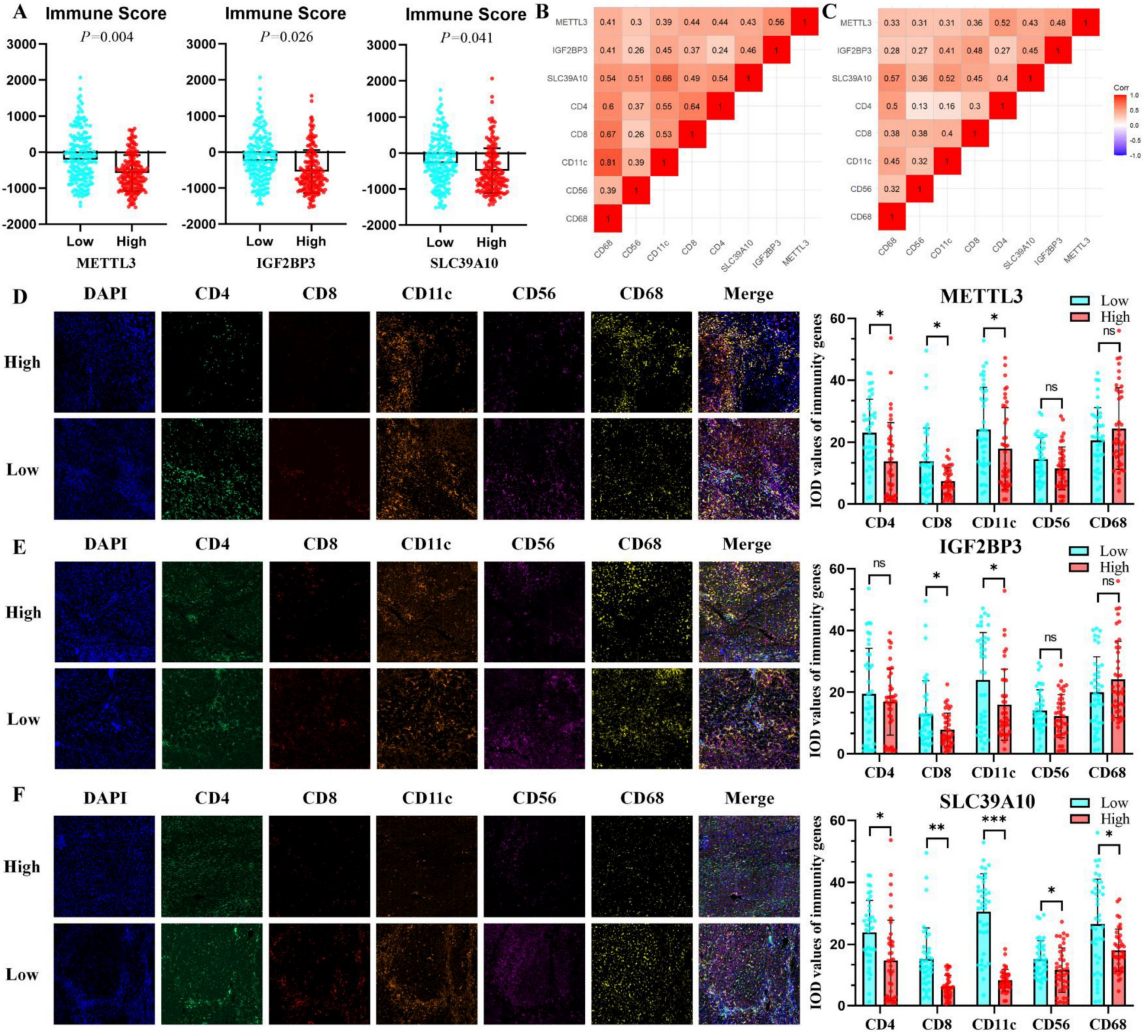
** METTL3-IGF2BP3-SLC39A10 was correlated with HCC immune microenvironment.** In TCGA-LIHC dataset, Analysed correlation of METTL3-IGF2BP3-SLC39A10 with HCC immunity scores (A), the correlation METTL3, IGF2BP3, SLC39A10 with main immune cell in TCGA-LIHC (B) and our center cohort (C), Investigating the effect of METTL3 (D), IGF2BP3 (E) and SLC39A10 (F) on the immune microenvironment in HCC. *p < 0.05. **p < 0.01. ***p < 0.001. NS, no significance.

The high-glucose microenvironment of tumors not only affects tumor cell function but also plays a crucial role in immune cell infiltration within the tumor microenvironment 28. Additionally, the high expression of SLC39A10, regulated by METTL3-IGF2BP3 in the high-glucose environment, promotes Zn ion uptake by HCC cells, which is essential for immune cell infiltration and function 29. To explore the effects of METTL3, IGF2BP3, and SLC39A10 on immune cell infiltration in HCC, we first analyzed their immunity scores in the TCGA-LIHC database. The results indicated a significant correlation between the expression of METTL3, IGF2BP3, and SLC39A10 with HCC immunity scores (Fig. 7A). We then examined the relationship between METTL3, IGF2BP3, and SLC39A10 with highly expressed immune cell populations (CD4, CD8, CD11C, CD56, and CD68) in liver cancer. The results demonstrated significant correlations between these genes and multiple immune cell expression levels (Fig. 7B). To further verify the correlation between METTL3, IGF2BP3, and SLC39A10 with immune cells, we labeled the immune microenvironment of HCC using multiple immunofluorescence staining techniques on serial sectioned HCC tissues (Fig. 7C). It should be noted that the xenograft model of nude mice in this study was not used to verify the phenotype of immune cell infiltration due to the lack of functional T cells.

We found that the abundance of CD4+, CD8+, and CD11C+ cells infiltrating the METTL3 high-expression group was significantly lower than that in the METTL3 low-expression group (Fig. 7D). The number of CD8+ and CD11C+ cells was also significantly reduced in hepatocellular carcinoma tumor tissues in the IGF2BP3 high-expression group (Fig. 7E). SLC39A10 expression was significantly different from the expression of CD4, CD8, CD11C, CD56, and CD68 (Fig. 7F). Overall, our results demonstrate a close association between METTL3-IGF2BP3-SLC39A10 and the infiltration of immune cells in the tumor microenvironment. As shown by the statistical results of multiple fluorescence staining described above, METTL3, IGF2BP3, and SLC39A10 consistently affected the infiltration of CD8+ cells in the tumor microenvironment. Additionally, we analyzed the prognostic value of immune cells. The results showed that CD4, CD8, CD11c, CD56, and CD68 were all associated with OS in HCC, whereas only CD4 and CD11c correlated with DFS (Fig. S5B).

Furthermore, we performed tumorigenesis assays in C57BL/6 mice, which were divided into five groups: Sh-NC + Vector, Sh-NC + Sh-SLC39A10, Sh-NC + SLC39A10, Sh-METTL3 + SLC39A10, and Sh-IGF2BP3 + Ov-SLC39A10. Multiplex immunofluorescence staining was performed on the subcutaneous tumors, with CD56 (human NK cell marker) replaced by NCR1 (murine NK cell marker). The results demonstrated that SLC39A10 promoted subcutaneous tumorigenesis of HCC in C57BL/6 mice and inhibited immune infiltration in the tumor microenvironment; conversely, knockdown of METTL3 or IGF2BP3 rescued the pro-tumorigenic and immune-suppressive effects induced by SLC39A10 overexpression (Fig. S5C). These findings collectively reveal the relationship between METTL3-IGF2BP3-SLC39A10 and the microenvironment of HCC and immune cell expression.

## Discussion

Disturbances in the body's glucose metabolism, particularly the persistent high-glucose extracellular environment associated with diabetes mellitus, are linked to the malignant progression of many tumors 4. However, the molecular mechanisms by which tumor cells sense extracellular glucose and transmit high-glucose signals intracellularly remain unclear. m^6^A methylation, the most widespread RNA methylation modification, plays a crucial role in tumor glucose metabolism 30, 31. Yet, it remains unexplored whether m^6^A can function as a cellular receptor to sense glucose levels in the extracellular matrix and transmit extracellular hyperglycemic signals. In this study, we propose a novel hypothesis that METTL3-IGF2BP3 acts as a glucose-binding regulatory module in HCC cells, sensing the extracellular high-glucose environment, transmitting high-glucose signals, and promoting glycolysis. Furthermore, we elucidate the mechanism by which METTL3-IGF2BP3 activates the PI3K-AKT pathway to promote the proliferation and glycolysis of HCC cells. These findings offer new insights and potential targets for the prevention and treatment of HCC.

Our study revealed that m^6^A levels were significantly elevated in HCC patients with hyperglycemia compared to those with normal blood glucose. This elevated level of RNA m^6^A methylation modification was primarily mediated by METTL3, a key regulator of m^6^A modification in various tumorigenesis processes 9, 32. By analyzing major pathways and transcription factors related to glucose metabolism in HCC cells, we identified the activation of the PI3K-AKT pathway in the extracellular high-glucose environment. Additionally, the expression level of c-MYC, a transcription factor regulated by PI3K-AKT, was also significantly elevated and mediated METTL3 transcription. These findings suggest that HCC cells exposed to a high-glucose environment can directly enhance m^6^A methylation levels by promoting METTL3 transcription.

RNA m^6^A methylation modification is a regulatory network composed of “writers”, “readers”, and “erasers” 33. We analyzed changes in “readers” in a high-glucose environment and found that IGF2BP3, an RNA-binding protein with diverse roles in tumor glucose metabolism, was significantly and consistently elevated. For instance, IGF2BP3 deubiquitination promotes endometrial cancer progression and glycolysis 34. IGF2BP3's recognition of PDK4 m^6^A enhances its mRNA stability and translation, promoting glycolysis in HCC cells 11. Recent studies have demonstrated that glucose can act as a signaling molecule, interacting with proteins, especially RNA-binding proteins 13. Based on these findings, we hypothesized that IGF2BP3, as an RNA-binding protein, could directly bind to glucose to transmit glucose signaling within the cell. Our results confirmed that glucose can bind to the RRM1-2 and KH3-4 structural domains of IGF2BP3. Furthermore, binding to the KH3-4 structural domain promotes IGF2BP3's recognition of RNA m^6^A modification. These experimental results unveil a novel mechanism whereby glucose regulates m^6^A modification independently of glucose metabolism.

Zn ions, an essential trace element for cell survival, play a crucial role in tumor cell proliferation, metabolism, and maintenance of the immune microenvironment 29, 35. Cellular uptake and efflux of Zn ions are primarily mediated by Zn ion transporter proteins, which can be influenced by glucose levels 36, 37. Takatani-Nakase et al. 38 observed that breast cancer cells increase their uptake of Zn ions in a high-glucose environment. However, the molecular mechanisms underlying tumor cell promotion of glucose uptake in response to high extracellular glucose levels require further investigation. Our study demonstrated that a high-glucose extracellular environment could promote METTL3 transcription and elevate IGF2BP3 recognition of m^6^A modification sites in HCC cells. This, in turn, promoted the stability and expression of SLC39A10, a Zn ion transporter responsible for transporting extracellular Zn ions into the cell in an m^6^A-dependent manner. These findings establish a connection between glucose sensing and Zn ion transport, expanding our understanding of the regulation of intra- and extracellular Zn ions by HCC cells.

It is well-established that the uptake of Zn ions by HCC cells not only activates various metalloproteinases but also induces the activation of certain signaling pathways, including the MAPK/ERK and PI3K/AKT pathways 39, 40. These pathways are significantly activated by HCC cells in a high-glucose culture environment. We hypothesized that SLC39A10-mediated Zn ion influx might affect key enzymes activating the PI3K-AKT pathway. By intersecting Zn ion-related proteins and PI3K-AKT pathway-related proteins from GeneCard, we identified ADAM17, which was consistently expressed with SLC39A10. ADAM17 is a highly Zn ion-dependent metalloprotease that cleaves and releases multiple epidermal growth factor receptor (EGFR) ligands, activating the EGFR-PI3K-AKT signaling pathway and promoting tumor proliferation and glucose metabolism 41, 42. To verify the role of Zn ions in activating the PI3K-AKT pathway, we directly regulated the extracellular Zn ion concentration and competitively inhibited the binding of Zn ions to ADAM17 using TAPI. These experiments support the role of Zn ions in PI3K-AKT pathway activation.

The tumor's high-glucose microenvironment and the imbalance of intra- and extracellular zinc ions not only affect the tumor's signaling pathways but also irreversibly impact a wide range of cells in the tumor microenvironment 43, 44. Li et al. 45 observed that reduced zinc concentration in the interstitial matrix induces a “zinc-deficient” state in immune cells, promoting tumor immune evasion. Beyond validating the regulatory relationship between METTL3-IGF2BP3-SLC39A10 in activating the PI3K-AKT pathway and glucose metabolism in HCC, we also analyzed the relationship between METTL3-IGF2BP3-SLC39A10 and HCC immunity. Our findings suggest that METTL3, IGF2BP3, and SLC39A10 are all significantly correlated with HCC immune levels, particularly SLC39A10, which is significantly and negatively correlated with CD4, CD8, CD11C, CD56, and CD68 expression. Moreover, METTL3-IGF2BP3-SLC39A10 were consistently negatively associated with the infiltration of CD8+ T cells in the HCC microenvironment. As well-established, CD8+ T cells play a crucial role in anti-tumor immunity in hepatocellular carcinoma. Our results indicated that METTL3-IGF2BP3-SLC39A10 could decrease the infiltration of immune cells, contributing to the formation of an immunosuppressive microenvironment. This provides novel insights into the relationship between METTL3-IGF2BP3-SLC39A10 and immune regulation in HCC.

Despite the novel insights of this study, several limitations should be noted for objective interpretation. First, the *in vitro* high glucose concentration (25 mM), while conforming to standard cell culture protocols, may exceed the actual tumor microenvironment glucose levels in clinical diabetic patients (typically 5-10 mM under poor control), potentially affecting the translational relevance of our observations. Future studies should validate the METTL3-IGF2BP3-SLC39A10 axis using clinically mimetic glucose concentrations. Second, the BALB/c nude mouse xenograft model lacks functional T cells and key immune components. Given the axis's regulatory role in immune cell infiltration (e.g., CD8⁺ T cells), it cannot fully recapitulate tumor-immune crosstalk in humans, necessitating humanized or patient-derived xenograft (PDX) models for further verification. Third, the clinical analysis is retrospective with a limited single-center sample size (80 paraffin-embedded and 16 fresh samples) and no external validation, which may introduce selection bias and limit generalizability. Multicenter, prospective studies with larger cohorts are needed to confirm the clinical utility of these molecules as prognostic biomarkers or therapeutic targets.

In conclusion, a high extracellular glucose environment enhances IGF2BP3's recognition and binding to RNA m^6^A modification sites and promoting METTL3 transcription in hepatocellular carcinoma cells. METTL3-IGF2BP3 subsequently promotes SLC39A10 expression and enhances Zn ion uptake by HCC cells in an m^6^A-dependent manner. Elevated intracellular Zn ion levels catalyze ADAM17 activity, activate the EGFR-PI3K-AKT pathway, and promote proliferation, glucose uptake, and glycolysis in HCC cells. These findings elucidate a novel molecular mechanism underlying HCC progression caused by dysglycemia, offering potential targets for the development of therapeutic inhibitors. Targeted drugs against METTL3/IGF2BP3^46^,^47^, combined with pathway inhibition, metabolic intervention, or immunotherapy, are expected to offer more hope for the treatment of HCC.

## Supplementary Material

Supplementary materials and methods, figures and tables.
