# Supplementary material for: METTL3-IGF2BP3 as a glucose sensor in hyperglycemic microenvironment promotes tumorigenesis and glycolysis
Source: Int J Biol Sci. 2026 Apr 16;22(9):4461–76. doi: 10.7150/ijbs.125135 (PMC13181852; doi:10.7150/ijbs.125135)
Supplement: Supplementary file 1 — Supplementary materials and methods, figures and tables. [file ijbsv22p4461s1.pdf]

Supplementary materials

Materials and Methods

Patients

In the present study, 80 matched paraffin samples of normal and tumor tissues were collected from patients with HCC who were diagnosed and underwent radical resection at the Second Affiliated Hospital of Zhejiang University between January 2012 and December 2015. Additionally, fresh normal and tumor tissues from 16 HCC patients who underwent radical hepatectomy from January 2020 to December 2021 were collected and stored at -80°C. Patients who died within 3 months of surgery or had multiple primary tumors were excluded. All patients received no preoperative chemotherapy or radiotherapy. Tissue sections were reviewed by experienced pathologists from the Second Affiliated Hospital of Zhejiang University. Written informed consent was obtained from all patients, and the study adhered to the guidelines of the Ethics Committee of the Second Affiliated Hospital of Zhejiang University (Ethics No: 2024-0397).

The definition of “hyperglycemia group” in this study refers to the Guidelines for the Prevention and Treatment of Type 2 Diabetes Mellitus in China (2020 Edition) and the WHO Diagnostic Criteria for Diabetes Mellitus, with specific criteria as follows: ① Confirmed type 2 diabetes mellitus (T2DM): fasting blood glucose (FBG) ≥7.0 mmol/L, or glycated hemoglobin (HbA1c) ≥6.5%, or 2-hour postprandial blood glucose ≥11.1 mmol/L; ② Impaired glucose tolerance (IGT): FBG 5.6~6.9 mmol/L and 2-hour postprandial blood glucose 7.8~11.0 mmol/L. The “non-hyperglycemia group” was defined as patients with FBG <5.6 mmol/L, HbA1c <5.7%, and no history of diabetes or impaired glucose tolerance (see Supplementary Table S1 for detailed baseline data).

Cell culture

The human hepatocellular carcinoma cell lines Hep3B and Huh7, along with the human embryonic kidney cell line 293T, were obtained from the cell bank of the Shanghai Institutes for Life Sciences, Chinese Academy of Sciences. These cell lines were recently authenticated by short tandem repeat (STR) profiling and were rigorously tested for mycoplasma contamination and cell viability.

The cells were cultured in a humidified incubator at 37°C with 5% CO_2. They were maintained in Dulbecco's Modified Eagle Medium (DMEM; Gibco, Carlsbad, CA, USA) supplemented with 10% fetal bovine serum (FBS), 100 units/mL penicillin, and 100 μg/mL streptomycin. All cell lines were confirmed to be negative for mycoplasma contamination using the MycoAlert™ Mycoplasma Detection Kit (Lonza, LT07-418).

Human hepatocellular carcinoma (HCC) cell lines (Hep3B, Huh7) and the human embryonic kidney cell line 293T were routinely cultured in Dulbecco's Modified Eagle Medium (DMEM)-based medium, supplemented with 10% fetal bovine serum (FBS; Gibco, Cat. No. 10099141), 100 U/mL penicillin, and 100 μg/mL streptomycin (Sigma-Aldrich, Cat. No. P4333) at 37°C in a humidified incubator with 5% CO_2.

To accurately mimic physiological and pathological glycemic microenvironments, three gradient glucose concentration systems were established by mixing high-glucose DMEM and glucose-free DMEM at specific volume ratios. The final glucose concentration of each group was verified using a glucose assay kit to ensure an error of < 5%:

Low-glucose (LG) group: Final glucose concentration of 5 mmol/L. Prepared by mixing high-glucose DMEM (glucose concentration: 4500 mg/L, equivalent to 25 mmol/L; Gibco, Cat. No. 11965092) and glucose-free DMEM (no glucose added; Gibco, Cat. No. 11966025) at a volume ratio of 1:4. This concentration simulates the fasting physiological blood glucose range (3.9–6.1 mmol/L) in healthy individuals.

Medium-glucose (MG) group: Final glucose concentration of 15 mmol/L. Prepared by mixing high-glucose DMEM and glucose-free DMEM at a volume ratio of 3:2. This concentration mimics the postprandial mild hyperglycemia fluctuation range in patients with prediabetes or poorly controlled blood glucose.

High-glucose (HG) group: Final glucose concentration of 25 mmol/L. Commercial high-glucose DMEM was used directly (no mixing required), which simulates the tumor-localized hyperglycemic microenvironment in diabetic patients with poorly controlled blood glucose and is consistent with the standard concentration of high-glucose models in cell culture.

All mixed media were sterile-filtered through a 0.22 μm membrane (Millipore, Cat. No. SLGP033RB), and the final glucose concentration was verified using a glucose oxidase assay kit (Thermo Fisher, Cat. No. A22189) to ensure accuracy and stability across groups. After 24 h of adaptive culture following cell seeding, the medium was replaced with the aforementioned gradient

glucose concentration media. Cells were further cultured for 48–72 h before being used for subsequent functional experiments (e.g., CCK-8 proliferation assay, glucose uptake assay, Seahorse extracellular acidification rate/oxygen consumption rate analysis, Western blot, and qPCR detection).

RNA extraction and real-time PCR

Total RNA was isolated using TRIzol reagent following the manufacturer's protocol. Reverse transcription was conducted with PrimeScript RT Enzyme (Takara Biotechnology). Real-time PCR amplification was carried out on a QuantStudio 5 Real-time PCR System using SYBR® Premix Ex Taq™ II. Cycling conditions were as follows: 95°C for 10 seconds, followed by 40 cycles of 95°C for 5 seconds and 60°C for 31 seconds. Relative gene expression was calculated using the 2-ΔΔCT method, with β-Actin serving as the internal control. Primer sequences are listed in Supplementary Table S1.

β-actin (Gene ID: NM_001101.5) was selected as the reference gene for qPCR experiments, with the following justifications: Biological rationality: β-actin is a core component of the eukaryotic cytoskeleton and a housekeeping gene in hepatocellular carcinoma (HCC) cell lines (Hep3B, Huh7). Its transcriptional level is not significantly affected by glucose concentrations (low glucose: 5 mmol/L, medium glucose: 15 mmol/L, high glucose: 25 mmol/L), cell proliferation status, or metabolic microenvironment changes, which meets the MIQE core principle that "reference genes should be stably expressed and unrelated to experimental treatments". Experimental consistency: β-actin has been used as the internal reference for protein level detection in Western blot experiments of this study. Using the homologous reference gene for qPCR can reduce systematic errors between different experimental techniques and improve data correlation.

To ensure the expression stability of the reference gene under the experimental conditions, the following validations were performed in accordance with MIQE guidelines: Experimental design: Hep3B and Huh7 cells treated with low glucose (LG), medium glucose (MG), and high glucose (HG) for 48 h were collected (3 biological replicates per group, 3 technical replicates per biological replicate). Total RNA was extracted and reverse-transcribed into cDNA. The Ct values of β-actin were detected using the same qPCR reaction system as the target genes (PrimeScript RT Master Mix, Takara, Cat. No. RR036A; TB Green Premix Ex Taq II, Takara, Cat. No. RR820A). Stability analysis tools: The expression stability of β-actin was analyzed using GeNorm (v3.5) and NormFinder

(v0.953), two commonly used software for qPCR reference gene validation: GeNorm analysis: The expression stability parameter (M-value) of β-actin in each group was calculated. An M-value < 1.5 is the MIQE-recommended standard for "stable expression". In this study, the M-values of β-actin in Hep3B cells (LG, MG, HG groups) were 0.28±0.05, and those in Huh7 cells were 0.31±0.04, both far below 1.5, indicating no significant fluctuations in its expression. NormFinder analysis: The stability value of β-actin was calculated (lower values indicate higher stability). The stability values of β-actin in Hep3B and Huh7 cells were 0.062 and 0.071, respectively, both in the "highly stable" range (< 0.1).

Validation of primer specificity: The specificity of β-actin primers was confirmed by melting curve analysis: a single melting peak (Tm value: 85.2±0.3°C) was generated after qPCR, with no non-specific peaks or primer dimer peaks. Additionally, agarose gel electrophoresis verified that the amplified product was a single target band (180 bp, consistent with the expected fragment size), eliminating the interference of non-specific amplification.

The relative expression levels of all target genes (e.g., METTL3, IGF2BP3, SLC39A10) were calculated using the $2^{(-\Delta\Delta Ct)}$ method, with the normalization process as follows: Calculate the Ct value difference between the target gene and the reference gene (β-actin) in each sample: ΔCt = Ct (target gene) - Ct (β-actin). Use the low-glucose group (LG) as the calibrator, and calculate the $\Delta\Delta Ct$ value of each treatment group (MG, HG) relative to the calibrator: $\Delta\Delta Ct$ = ΔCt (treatment group) - ΔCt (calibrator). Relative expression level of the target gene = $2^{(-\Delta\Delta Ct)}$. The results are presented as "mean±SD", with at least 3 biological replicates per group to ensure data compliance with the reproducibility requirements of MIQE guidelines.

Western blot

HCC tissue or cell samples were lysed with RIPA buffer in an ice bath for 30 minutes. Cell lysates were quantified using the BCA Protein Detection Kit (Promega) and separated by 4-20% SDS-PAGE electrophoresis. The proteins were then transferred to PVDF membranes (Millipore, USA). The membranes were blocked with 5% skimmed milk at 37°C for 1 hour and subsequently incubated with primary antibodies against METTL3 (1:1000), IGF2BP3 (1:1000), FLAG (1:3000), SLC39A10 (1:1000), ADAM17 (1:1000), β-actin (1:5000), phospho-Akt (Ser473) (1:2000), Akt (1:2000), EGFR (1:4000), and phospho-EGFR (1:4000). All primary antibodies were obtained from Proteintech (China) except for SLC39A10, which was from GeneTex (USA). Secondary antibodies,

HRP-conjugated Affinipure goat anti-mouse IgG and HRP-conjugated Affinipure goat anti-rabbit IgG (both at 1:5000), were used to detect primary antibody binding. The chemiluminescent signal was captured using a Tanon4600Western Chemiluminescent Gel Imaging System (Shanghai Tianneng Technology Co., Ltd.) and analyzed with Image Lab 6.0.1 (Bio-Rad, USA).

Cell colony formation assay

In the cell colony formation assay, Hep3B and Huh7 cells were cultured in 6-well plates at a concentration of 800 cells/well. Then the medium was changed every two days and the cells were allowed to grow for a fortnight. The cells were washed with PBS and fixed, and stained with 1% crystal violet solution for 15 min. The excess stain was rinsed with PBS, and then the images were captured with a microscope.

Cell counting kit-8 assay

HCC cells of each designated nomenclature group were transferred into 96-well plates (1×10^3 cells/well), and cell counting kit-8 (CCK-8) solution (beyotime, CO048M, China) was added to each well at 24, 48, 72 and 96 h, respectively, followed by 3 h of incubation. The absorbance of each well was recorded at 450 nm using a microplate reader (BioTek, Winooski, VT, USA).

Seahorse Metabolic Analysis

Extracellular acidification rate (ECAR) and oxygen consumption (OCR) were determined using the Seahorse XF Glycolytic Stress Test Kit and the Seahorse XF Cellular Mitochondrial Stress Test Kit (Agilent Technologies, Palo Alto, CA), respectively. Transfected Hep3B and Huh7 cell cells were cultured into hippocampus-specific 96-well cell culture plates overnight. Glucose, oligomycin, and 2-deoxyglucose (2-DG) were sequentially added to the corresponding wells of the sensor cassette for ECAR measurement. Oligomycin, SCCP antimycin A, and rotenone were sequentially added to the corresponding wells for OCR measurement. The prepared cell plates were then analyzed using a Seahorse XFe96 analyser (Agilent Technologies, Santa Clara, USA), and the cell culture plates were analyzed using Seahorse Wave software (Agilent Technologies, Santa Clara, USA) to analyze the data.

For the Seahorse XFe96 microplate (a common model for glucose metabolism detection), the

seeding density of Hep3B/Huh7 cells was set to 1.5×10^4–3×10^4 cells per well. Prior to the formal experiment, a density gradient of 1×10^4, 2×10^4, and 3×10^4 cells per well was established to detect basal oxygen consumption rate (OCR)/extracellular acidification rate (ECAR) and ECAR changes after glucose stimulation. The optimal density was selected based on the criteria of "signal-to-background ratio ≥ 5 and ECAR < 90 pmol/min", and 2×10^4 cells per well was ultimately determined as the cell density for the cell energy metabolism assay.

Hep3B/Huh7 hepatocellular carcinoma cells highly express glucose transporters (GLUTs) and exhibit active metabolism. A medium-to-high concentration of 20-40 μM (for 2-NBDG, a fluorescently labeled glucose analog) was chosen to avoid insufficient fluorescent signals caused by low concentrations (<10 μM) and non-specific cytotoxicity induced by high concentrations (>50 μM). For preliminary experimental validation, a concentration gradient of 10, 20, 30, and 40 μM was established, and fluorescence intensity was detected at an excitation wavelength of 488 nm and an emission wavelength of 530 nm. The lowest concentration at which "the fluorescence signal reached a plateau with increasing concentration" was selected, and 20 μM was ultimately confirmed as the optimal concentration of 2-NBDG.

The incubation time for 2-NBDG was set to 30-60 minutes. Both Hep3B/Huh7 cells and H9c2 cells are characterized by high GLUT expression. Effective uptake of 2-NBDG could be achieved within 30 minutes, while extending the incubation time to 60 minutes further enhanced the fluorescent signal. Incubation was performed in a 37°C, 5% CO_2 incubator. After incubation, cells were washed twice with pre-cooled 1×PBS to terminate the uptake reaction, and fluorescence detection was completed within 30 minutes to prevent signal attenuation.

To verify the specificity of glucose uptake, 10 mM D-glucose was added (co-incubated with 2-NBDG). By competing for GLUT binding sites, D-glucose reduced 2-NBDG uptake. A reduction in fluorescence intensity by ≥40% compared to the experimental group indicated specific 2-NBDG uptake. The negative control consisted of cells not incubated with 2-NBDG (to correct for autofluorescence).

The Seahorse XF96 analyzer was used to measure the oxygen consumption rate and extracellular acidification rate. Equal numbers of cells were seeded into each well. After the experiment, total protein content in each well was determined using the BCA assay, and all OCR and ECAR data were normalized based on the total protein content of the corresponding samples. Cell glucose uptake capacity was measured using the 2-NBDG fluorescence assay. After the experiment, total protein

content in the samples was determined using the BCA method. Fluorescence intensity values were normalized to total protein content to calculate relative glucose uptake levels.

Biotin pull-down assay

The biotin-glucose pull-down assay was performed based on previous studies[12, 14]. Briefly, Magic Dynabeads MyOne Streptavidin T1 (Thermal Fisher) was pre-incubated with free biotin or biotin-labeled glucose for 30 min at RT, and then rotationally incubated with cell lysates, recombinant proteins, or peptides overnight at 4 degrees. Beads were rinsed 4 times with PBS and then analyzed by immunoblotting. The detailed experimental procedures:

(1) Preparation of Biotin-Labeled Glucose-Streptavidin-MagBead Complex

Transfer 20 μL of <3> Streptavidin-MagBeads into an EP tube, place it on a magnetic stand, and let it stand for 30 s to adsorb and collect the MagBeads; discard the supernatant. Add 500 μL of pre-cooled <1> 1×PBS, gently pipette to resuspend the MagBeads, adsorb on the magnetic stand, and discard the supernatant; repeat the washing twice. Add 100 μL of pre-cooled <1> 1×PBS to the washed MagBeads, followed by 100 pmol of <7> biotin-labeled glucose. Incubate with gentle rotation at room temperature (25°C) for 30 min to ensure sufficient binding between biotin and streptavidin on the MagBead surface. Adsorb the MagBeads on the magnetic stand, discard the supernatant, and resuspend the MagBeads with 100 μL of pre-cooled <2> cell lysis buffer; set aside for later use.

(2) Protein Extraction (Optimized Based on Original CoIP Procedures)

Washing: Wash the samples (approximately 2×10^7 Hep3B/Huh7 cells) twice with 1 mL of pre-cooled <1> 1×PBS; blot dry the 1×PBS completely after the last wash. Lysis: Add 1 mL of <2> cell lysis buffer and 10 μL of <21> protease inhibitor cocktail; lyse gently on ice for 20–30 min (gently invert and mix 3 times during lysis to avoid violent lysis that would disrupt glucose-IGF2BP3 binding). Centrifugation: Centrifuge at 4°C, 13000 g, for 15 min; collect the supernatant into a new EP tube, which is the total protein lysate.

(3) Grouped Incubation (Supplemented with Competitive Control, 4 Groups in Total)

Take the total protein lysate and treat it according to the following groups (3 technical replicates set for each group): Input Group: Take 100 μL of lysate, add 25 μL of 5×SDS loading buffer, and store at -20°C for later use. Experimental Group (Biotin-Glucose Group): Take 900 μL of lysate, add 100 μL of the "biotin-labeled glucose-MagBead complex" prepared in Step (1). Blank Control Group (Free

Biotin Group): Take 900 μL of lysate, add 100 μL of the "100 pmol free biotin + streptavidin-MagBead" complex (to rule out non-specific binding of biotin itself). Competitor Group: Take 900 μL of lysate, add 100 μL of the "100 pmol biotin-labeled glucose + 1 mmol/L <8> non-biotin-labeled glucose + MagBead" complex (the concentration of non-labeled glucose is 10^4-fold that of biotin-labeled glucose, designed based on the competitive inhibition experiment in [3,4]). Place all groups on a vertical shaker at 4°C and incubate with gentle shaking for 12–16 h (overnight) to ensure sufficient binding between IGF2BP3 and biotin-labeled glucose (referring to the glucose-NSUN2 incubation strategy in [3]).

(4) MagBead Washing

After incubation, place the EP tubes of all groups on a magnetic stand, let them stand for 1 min to adsorb and collect the MagBeads, and discard the supernatant. Add 1 mL of pre-cooled <4> Wash Buffer I, gently pipette to resuspend the MagBeads, shake on a vertical shaker at 4°C for 5 min, adsorb on the magnetic stand, and discard the supernatant; repeat the washing 4 times. Add 1 mL of pre-cooled <5> Wash Buffer II, shake on a vertical shaker at 4°C for 5 min, adsorb on the magnetic stand, and discard the supernatant; perform 1 wash (the high-salt buffer further removes non-specifically bound contaminating proteins, referring to the small molecule-protein interaction washing protocol in [1,6]). After the last wash, blot dry the residual buffer and retain the MagBead pellet.

(5) Protein Elution

Add 60 μL of <6> Elution Buffer to the MagBeads of each group, and gently pipette to resuspend. Denature and elute in a boiling water bath for 10 min, then immediately place on ice to cool. Adsorb the MagBeads on the magnetic stand, collect the supernatant into a new EP tube, and store at -20°C for later use.

(6) Detection and Analysis

Take 16 μL of the eluted supernatant (from the Experimental Group and Control Groups) and 16 μL of the Input Group sample; add 4 μL of 5×SDS loading buffer to each, and incubate in a boiling water bath for 5 min. Perform SDS-PAGE electrophoresis (10% separating gel), transfer the proteins to a PVDF membrane, block the membrane, then add the primary antibody against IGF2BP3 (1:1000 dilution) and incubate overnight at 4°C. After incubating with the secondary antibody, develop the membrane and analyze the band grayscale of IGF2BP3 in each group: A clear band should be observed in the Experimental Group, while the band in the Blank Control Group should be very weak

or absent, and the band grayscale of the Competitor Group should be reduced by ≥70% compared to the Experimental Group.

RNA stability

To measure the stability of RNA in IGF2BP3 stable knockout or control Hep3B and Huh7 cells, 5 μg/ml of actinomycin D (MCE, USA) was added to the cells, and the cells were collected after incubation for the indicated times (0, 1, 2, 4, and 8 h), and RNA was isolated from these cells for qRT-PCR analysis.

Chromatin immunoprecipitation assay

Chromatin immunoprecipitation (ChIP) assays were performed using the Magna ChIP Kit (Millipore, Bedford, MA, USA) according to the manufacturer's protocol. Hep3B and Huh7 cells were treated with formaldehyde to produce DNA protein crosslinks. The cell lysates were then sonicated to produce 200-300 bp chromatin fragments and the lysates were immunoprecipitated with c-MYC antibody (CY5150, abways, China) or IgG as a control. The precipitated chromatin DNA was recovered and determined by qPCR. Primer sequences are shown in Supplementary Table S2 (hyperlink).

Luciferase reporter assay

After seeding three times in 24-well plates and allowing to stand for 24 h, cells were transfected with 200 ng of signaling luciferase reporter gene (OriGene) + 5 ng of pRL-TK kidney reporter plasmid and siRNA (siNC or siSLC39A10) or pGL-SLC39A10 (wt, mut1 or mut2) plasmid using the Liposome 3000 reagent (L3000001, Thermo, USA). Dual luciferase reporter gene assay was performed 48 hours after transfection using the Dual-Luciferase Reporter Gene Assay Kit (E1910, Promega, USA).

Immunofluorescence

Preserved HCC tissue and adjacent normal embedded tissue samples in paraffin were serially sectioned to a thickness of 5 μm. m6A, METTL3, IGF2BP3, and SLC39A10 antibodies (antibody

information has been presented previously) were then applied to the slices, and the slices were then incubated with Alexa Fluor 488 Anti-Rabbit Immunoglobulin diluted at a ratio of 1:500 (#4412,1:500, CST), Alexa Fluor 594 anti-mouse immunoglobulin (#8890,1:500, CST), and the nuclei were stained with DAPI and incubated for 20 min at 37 °C. Image acquisition was performed using a Zeiss LSM-710 confocal microscope to observe the sections. The criteria for staining results were as follows: 5 tumor regions were randomly selected for protein expression under the microscope. Two pathologists were blinded to any differences to avoid bias.

Hematoxylin-Eosin (H&E) Staining Pathological Analysis

Protocol: Deparaffinization: Place 5 μm-thick paraffin-embedded tumor tissue sections into xylene I and xylene II sequentially, incubate for 5~10 minutes each to completely remove paraffin; Graded Hydration: Hydrate the sections through a graded ethanol series: 100% ethanol, 95% ethanol, 80% ethanol, 70% ethanol, 3~5 minutes per step, and finally rinse with distilled water 3 times, 1 minute each time; Hematoxylin Staining: Immerse the hydrated sections in hematoxylin staining solution (Sigma-Aldrich, Cat. No. H9627) and incubate at room temperature for 5~10 minutes to stain cell nuclei; Washing to Remove Excess Dye: Rinse the sections with running distilled water for 5 minutes to remove unbound dye; Differentiation: Immerse the sections in 1% hydrochloric acid-ethanol differentiation solution (1% v/v HCl + 75% ethanol) for 10~30 seconds until the sections turn pale blue-purple, then quickly rinse with distilled water to terminate differentiation; Bluing Reaction: Soak the sections in 0.5% ammonia water bluing solution (0.5% v/v $NH_3 \cdot H_2O$) for 5 minutes to clarify the blue color of cell nuclei, followed by rinsing with distilled water 3 times, 1 minute each time; Eosin Staining: Immerse the sections in eosin staining solution (Sigma-Aldrich, Cat. No. E4009) and incubate at room temperature for 2~5 minutes to stain cytoplasm; Dehydration and Clearing: Dehydrate the sections through a graded ethanol series: 70% ethanol, 80% ethanol, 95% ethanol, 100% ethanol, 3~5 minutes per step, then clear in xylene I and xylene II for 5~10 minutes each; Mounting and Observation: Add a drop of neutral balsam (Solarbio, Cat. No. G8590) to the center of the section, cover with a coverslip to avoid air bubbles, air-dry at room temperature, and observe the pathological morphology of tumor tissues (e.g., proliferative activity, necrosis degree, stromal infiltration) under a light microscope.

To avoid subjective bias, both "tumor weighing" and "hematoxylin-eosin (H&E) staining

pathological analysis" were performed using a double-blind method in this study. The detailed procedures are as follows:

1. Double-Blind Operation for Tumor Weighing

Coding and Labeling: After the mice were euthanized, tumor samples from each experimental group were randomly coded by "non-experimental designers" (e.g., using letter-number combinations such as "A1, B3, C5"). The coding table was sealed and stored, and the experimental operators responsible for weighing were unaware of the group information corresponding to each code. Weighing and Recording: The operator weighed the tumors using an electronic balance (accuracy: 0.001 g) and only recorded the "code-weight" corresponding data without associating it with specific groups. Decoding and Matching: After all samples were weighed, another independent individual retrieved the coding table, matched the "code-weight" data with the actual experimental groups, and generated the final statistical dataset.

2. Double-Blind Evaluation for H&E Staining Pathological Analysis

Section Coding: After tumor tissues were fixed with 4% paraformaldehyde and embedded in paraffin, pathological technicians randomly coded the sections (the coding rule was consistent with that for tumor weighing but unrelated to the weighing codes). The section labels only displayed the codes without any group information. Blinded Evaluation: Two experienced pathologists (who did not participate in the study design) were invited as independent assessors. Without knowing the section groups, they evaluated key indicators of tumor tissues in accordance with the Pathological Diagnosis Guidelines for Liver Cancer, including: ① Tumor cell proliferation activity (counting mitotic figures under high-power microscopy); ② Degree of tumor necrosis (calculating the percentage of necrotic areas relative to the total tumor area); ③ Tumor stromal infiltration. Consistency Verification: If the consistency of the evaluation results between the two pathologists exceeded 90%, the average value was taken as the final result. If the consistency was less than 90%, a third pathologist conducted a blinded review, and the final result was determined by the majority opinion. Decoding and Statistical Analysis: After all pathological evaluations were completed, "non-evaluators" retrieved the coding table, matched the data with group information, and performed statistical analysis.

Determination of m6A levels

The total m6A content in 200 ng aliquots of total RNA extracted from Hep3B and Huh7 cell

subclones was measured using the m6A RNA Methylation Quantification Kit (C11051-1, Ribio, China) according to the manufacturer's instructions. To measure m6A+ SLC39A10 mRNA levels, m6A immunoprecipitation was performed as previously described. A 1μg aliquot of m6A antibody was conjugated to protein A-agarose plasma (Millipore) and incubated at 4 °C overnight. A 100 μg aliquot of total RNA was incubated with the antibody in immunoprecipitation buffer (50 mM Tris HCl, 750 mM NaCl, and 0.5% Igepal CA-630) supplemented with an RNase inhibitor for 3 h at 4 °C, and then the RNA was eluted from the beads in 300 μL of elution buffer (5 mM Tris-HCl, 1 mM EDTA). HCl, 1 mM EDTA, and 0. 05% SDS) in 300 μL of elution buffer (5 mM Tris-HCl, 1 mM EDTA, and 0. 05% SDS) with 4.2 μL of Proteinase K was incubated at 50 °C for 1.5 h. The m6A+ RNA was then purified by phenol/chloroform extraction and analyzed by RT-qPCR. The nucleotide sequences of the primers used to detect m6A+ SLC39A10 mRNA were as follows: 5′-ATG CAA CCT GAA GAC GTG TG-3′ and 5′-GAG ATT GAC TGG ATG GGC AT-3′.

This kit adopts A "competitive ELISA" design, achieving m^6A quantification through "antigen-antibody specific binding" and "competitive binding signal amplification". The key mechanisms are as follows: Specific recognition basis: The kit contains horseradish peroxidase (HRP) -labeled anti-M ^6A monoclonal antibody. This antibody can only specifically bind to the N6-methyladenosine (m^6A) site on RNA molecules and does not cross-react with unmodified adenosine (A) or other RNA modifications (such as m^5C, m^1A), which is the core to ensure the specificity of the detection. Competitive binding logic: The 96-well plate solid-phase carrier is pre-coated with "m^6A modified RNA standard" (fixed concentration antigen). When the sample to be tested (200ng of total cell RNA in the original text) is co-incubated with HRP-labeled m^6A antibody, the m^6A modified RNA in the sample will compete with the pre-coated m^6A standard on the plate for antibody binding - the higher the m^6A content in the sample, the more antibodies will be bound. The less antibodies there are remaining to bind to the standard substances on the plate; Signal output and quantification: After incubation, free antibodies are removed by washing, and the substrate 3,3',5,5' -tetramethylbenzidine (TMB) is added. HRP catalyzes TMB to generate a colored product, and the absorbance is detected at 450 nm. The absorbance value is inversely proportional to the m^6A content in the sample: the lower the absorbance, the higher the m^6A content. By plotting A standard curve with the m^6A standard provided in the kit, the relative levels of m^6A in the total RNA of Hep3B and Huh7 cells can be calculated.

Intracellular Zn^{2+} Level Detection

Intracellular free Zn^{2+} levels were measured using the Zn^{2+}-specific cell-permeable fluorescent probe ZnAF-2 DA (GC26167, GLPBIO). Upon entering cells, ZnAF-2 DA is hydrolyzed by cytoplasmic esterases to form ZnAF-2F, which binds specifically to Zn^{2+} and emits fluorescence (exhibiting high selectivity for Zn^{2+} with a cross-reactivity of < 1% for Ca^{2+}, Mg^{2+}, and other ions). The detailed steps are as follows:

Probe Preparation: Dissolve ZnAF-2 DA powder in dimethyl sulfoxide (DMSO) to prepare a 10 mmol/L stock solution, which is stored at -20℃ in the dark. Before use, dilute the stock solution to the working concentration with serum-free medium, and add 0.02% Pluronic F-127 (for solubilization to prevent probe aggregation). Cell Pretreatment: Hepatocellular carcinoma (HCC) cells (Hep3B/Huh7) were seeded in confocal dishes and cultured in high-glucose (25 mmol/L) medium for 48 h (corresponding experimental groups: si-NC, si-SLC39A10, Vector, OE-SLC39A10). Probe Loading: Aspirate the old medium, add serum-free medium containing ZnAF-2 DA (final concentration: 4 μmol/L), and incubate at 37℃ with 5% CO_2 in the dark for 45 min (to ensure sufficient probe entry into cells and hydrolysis). Washing and Counterstaining: Wash the cells 3 times with pre-warmed HBSS buffer (5 min per wash) to thoroughly remove uninternalized free probe and reduce background fluorescence. Add Hoechst 33342 (final concentration: 1 μg/mL) and incubate in the dark for 10 min for nuclear counterstaining.

Image Acquisition: Images were captured using a Zeiss LSM 880 confocal microscope with uniformly set parameters. Excitation wavelength (Ex): 490 nm (specific excitation for ZnAF-2F); Emission wavelength (Em): 510 nm (fluorescence emission from the Zn^{2+}-ZnAF-2F complex); Nuclei (Hoechst): Excitation 350 nm / Emission 461 nm; Laser power: 18% (to avoid photobleaching); scanning step: 0.5 μm. For each sample, 5 random fields (200× magnification) were selected, and only cells in the tumor parenchymal region were imaged.

Quantitative Analysis: Fluorescence signals were quantified using ImageJ software, following these steps: ① Outline the cytoplasmic region of individual cells as the Region of Interest (ROI), excluding nuclei and intercellular spaces; ② Measure the Mean Fluorescence Intensity (MFI) of each ROI; ③ For each sample, calculate the average MFI of at least 50 cells across 5 fields to determine the relative Zn^{2+} levels in each group.

Tumor xenograft experiments

All animal experiments were approved by the Animal Research Committee of the Ethics Committee of the Second Affiliated Hospital of Zhejiang University (Ethical Approval Number: 2024-057). The experiments were conducted in strict accordance with the Guide for the Care and Use of Laboratory Animals (National Institutes of Health, NIH).

Establishment of Diabetic Model: Four-week-old BALB/c nude mice were purchased from Shanghai Slek Laboratory Animal Co., LTD. The mice were housed in a specific pathogen-free (SPF) environment with a temperature of 22-25℃, humidity of 50%-60%, and a 12-hour light/12-hour dark cycle. They had free access to standard chow (before modeling) and water. Streptozocin (STZ) was used to induce the diabetic model, and the preparation method was as follows:Under ice-bath and light-shielding conditions, STZ powder was completely dissolved in 0.1mol/L sterile sodium citrate buffer (pH=4.5) to prepare the STZ working solution. The working solution was freshly prepared and used immediately, and injection was completed within 30 minutes after preparation to prevent STZ degradation and inactivation in aqueous solution, which might affect the modeling effect. To enhance the sensitivity of pancreatic β-cells to STZ, the mice were fasted for 12 hours (water-deprived) before STZ injection. During fasting, the mice were housed individually to avoid mutual feeding and ensure consistent pretreatment effects. On Day 0 and Day 7 of modeling, intraperitoneal injection (i.p.) was performed on the pretreated mice: Before injection, the abdominal skin of the mice was disinfected with 75% ethanol to prevent infection; A 1mL sterile syringe was used, and the needle was inserted into the abdominal cavity at a 30-45° angle from the left or right side of the midabdominal line (avoiding internal organs), followed by slow injection of the STZ working solution; The STZ injection dose was 100mg/kg body weight (b.w.), and the injection volume for each mouse was accurately adjusted according to its weight (e.g., 0.2mL of working solution containing 2mg STZ for a 20g mouse). Six hours after injection, the mice were provided with 5%-10% glucose water for free drinking to prevent fatal hypoglycemia caused by transient insulin release during acute damage of pancreatic β-cells by STZ; Twenty-four hours after injection, glucose water supply was stopped, and normal drinking water was resumed to avoid interference of long-term high glucose intake with subsequent blood glucose monitoring results. Blood samples were collected from the mice via tail vein puncture, and fasting blood glucose levels were measured using an NB-loT blood glucose meter (produced in Hangzhou): The first measurement was performed 3 days after the second STZ injection

on Day 7, and subsequent remeasurements were conducted every 2 days; Mice with two consecutive blood glucose values > 300mg/dL were considered to have a successfully established diabetic model; mice with blood glucose not meeting the standard were excluded from subsequent experiments.

Establishment of Subcutaneous Hepatocellular Carcinoma Xenograft Model: The Huh7 human hepatocellular carcinoma cell line was used to construct cell strains stably transfected with specific intervention plasmids for each subgroup (e.g., Sh-NC, Sh-METTL3, Vector, METTL3). Before the experiment, the cells were cultured to the logarithmic growth phase, harvested by digestion with 0.25% trypsin, washed twice with sterile PBS, and adjusted to a concentration of 2×10^7 cells/mL. The cell suspension was kept on ice for later use (to avoid decreased cell viability). Four-week-old BALB/c nude mice with successfully established diabetic models (normal blood glucose nude mice could be used as controls in some experiments, depending on the experimental groups) were selected for subcutaneous injection of hepatoma cells: The injection site was the hind abdomen of the mice, and the skin was disinfected with 75% ethanol before injection; A 1mL sterile syringe was used, and the needle was inserted into the subcutaneous tissue at a 15° angle, followed by slow injection of 0.1mL cell suspension (containing 2×10^6 Huh7 cells). After injection, the injection site was gently pressed for 10 seconds to prevent leakage of the cell suspension; Each mouse was injected with cells only on one side of the hind abdomen and housed individually to avoid scratches on the injection site caused by mutual fighting. One week after injection, the long diameter (length) and short diameter (width) of the tumors were measured every 5 days using digital calipers; The tumor volume was calculated using the formula: Volume = (length × width^2)/2 (unit: mm^3). Data were recorded each time, and tumor growth curves were plotted; During monitoring, the mental state, food and water intake of the mice, and the presence of redness, swelling, or ulceration at the tumor site were observed. If severe abnormalities occurred (e.g., weight loss > 20%), the experiment was terminated promptly. Six weeks after injection, the mice were euthanized by cervical dislocation (in line with animal ethics requirements); The mice were dissected, and the subcutaneous tumor tissues were completely excised. The tumor weight was measured and recorded using an electronic balance; A portion of the tumor tissue was fixed in 4% paraformaldehyde solution for 24 hours, and subsequent hematoxylin-eosin (H&E) staining was performed to observe the pathological characteristics of the tumor tissue. The remaining tissue was stored in a -80℃ refrigerator or immediately used for protein/RNA extraction according to experimental needs.

To avoid subjective bias, in this study, both "tumor weighing" and "hematoxylin-eosin (H&E)

staining pathological analysis" were performed using a double-blind method. The specific process is as follows: Coding markers: After the mice were euthanized, the tumor samples of each experimental group were randomly coded by "non-experimental designers" (such as using a combination of letters and numbers, like "A1, B3, C5"). The coding tables were sealed and kept, and the experimental operators (responsible for weighing) were unaware of the group information corresponding to the codes. Weighing record: The operator uses an electronic balance (with an accuracy of 0.001g) to weigh the tumor, only recording the corresponding data of "code - weight", without associating with groups. Decoding and matching: After all samples have been weighed, another independent person retrives the encoding table, matches the "encoding - weight" data with the actual grouping, and forms the final statistical data set.

Section coding: After the tumor tissue is fixed with 4% paraformaldehyde and embedded in paraffin, the pathology technicians randomly code the sections (the coding rule is consistent with the tumor weighing and has no relation to the weighing code). The section label only shows the code and does not contain grouping information. Blind assessment: Two experienced pathologists (who did not participate in the design of this study) were invited as independent assessors. Without knowing the section grouping, they evaluated the key indicators of tumor tissues in accordance with the "Pathological Diagnosis Guidelines for Liver Cancer", including: ① Tumor cell proliferation activity (counting mitotic figures under high-power microscopy), ② Degree of tumor necrosis (calculating the percentage of necrotic areas to the total tumor area), ③ Tumor interstitial infiltration. Consistency verification: If the consistency of the assessment results of two physicians is greater than 90%, the average value will be taken as the final result. If the consistency is less than 90%, a blind review will be conducted by a third pathologist, and the majority opinion will prevail in the end. Decoding statistics: After all pathological evaluations are completed, the "non-evaluators" retrieve the encoding table, match the grouping information and conduct statistical analysis.

Glucose Concentration Treatment for Cell Culture

Human hepatocellular carcinoma (HCC) cell lines (Hep3B, Huh7) and human embryonic kidney cell line 293T were routinely cultured in Dulbecco's Modified Eagle Medium (DMEM)-based medium, supplemented with 10% fetal bovine serum (FBS; Gibco, Cat. No. 10099141), 100 U/mL penicillin, and 100 μg/mL streptomycin (Sigma-Aldrich, Cat. No. P4333) at 37°C in a humidified incubator with

5% CO_2.

To accurately mimic physiological and pathological glycemic microenvironments, three gradient glucose concentration systems were established by mixing high-glucose DMEM and glucose-free DMEM at specific volume ratios. The final glucose concentration of each group was verified by a glucose assay kit to ensure an error of < 5%: Low-glucose (LG) group: Final glucose concentration of 5 mmol/L. Prepared by mixing high-glucose DMEM (glucose concentration: 4500 mg/L, equivalent to 25 mmol/L; Gibco, Cat. No. 11965092) and glucose-free DMEM (no glucose added; Gibco, Cat. No. 11966025) at a volume ratio of 1:4. This concentration simulates the fasting physiological blood glucose range (3.9–6.1 mmol/L) in healthy individuals; Medium-glucose (MG) group: Final glucose concentration of 15 mmol/L. Prepared by mixing high-glucose DMEM and glucose-free DMEM at a volume ratio of 3:2. This concentration simulates the postprandial mild hyperglycemia fluctuation range in patients with prediabetes or poorly controlled blood glucose; High-glucose (HG) group: Final glucose concentration of 25 mmol/L. Commercial high-glucose DMEM was used directly (no mixing required), which simulates the tumor-localized hyperglycemic microenvironment in diabetic patients with poorly controlled blood glucose and is consistent with the standard concentration of high-glucose models in cell culture.

All mixed media were sterile-filtered through a 0.22 μm membrane (Millipore, Cat. No. SLGP033RB), and the final glucose concentration was verified using a glucose oxidase assay kit (Thermo Fisher, Cat. No. A22189) to ensure accuracy and stability across groups. After 24 h of adaptive culture following cell seeding, the medium was replaced with the aforementioned gradient glucose concentration media. Cells were further cultured for 48–72 h before being used for subsequent functional experiments (e.g., CCK-8 proliferation assay, glucose uptake assay, Seahorse extracellular acidification rate/oxygen consumption rate analysis, Western blot, and qPCR detection).

Isothermal Titration Calorimetry (ITC) Study Methods and Results

1. Materials and Methods

1.1 Reagents and Materials

Ligand reagents: D-glucose (purity ≥99.5%, Sigma-Aldrich, USA); D-mannitol (purity ≥99.0%, Merck, Germany). Crystalline water was removed by vacuum drying (60℃, 24 h) before use.

Buffer: The experimental buffer system was 20 mmol/L Tris-HCl (pH 7.4) containing 150

mmol/L NaCl. It was filtered through a 0.22 μm polyethersulfone membrane (Millipore, USA) and vacuum degassed at 4°C for 30 min to eliminate bubble interference.

Target protein: IGF2BP3 binds to the ligand in this study was expressed via a prokaryotic expression system (E. coli BL21 strain), purified by two-step chromatography including Ni-NTA affinity chromatography (GE Healthcare, USA) and gel filtration chromatography (Superdex 200 column). The final purity was verified to be ≥95% by SDS-PAGE, and the protein concentration was determined by the BCA method (Thermo Fisher Scientific, USA) to be 10 μmol/L.

1.2 Instruments and Experimental Conditions

Instrument: A MicroCal PEAQ-ITC isothermal titration calorimeter (Malvern Panalytical, UK) was used. Before the experiment, the instrument was performance-verified with standardized calibration reagents (e.g., hexokinase-glucose reaction system).

Experimental parameters: The experimental temperature was controlled at 25.0±0.1°C; the stirring speed was set to 750 r/min; the ligand stock solution (glucose/mannitol) concentration was 100 μmol/L (at a 10:1 ratio to the protein concentration, complying with the ITC ligand excess principle), loaded into a 200 μL titration syringe; the protein solution (10 μmol/L) was added to a 200 μL sample cell and equilibrated for 30 min before titration.

Titration procedure: A total of 25 injections were performed. The first injection volume was 0.4 μL (to eliminate needle surface adsorption interference, data not included in analysis), and the subsequent 24 injections were 8 μL each; the interval between adjacent injections was 180 s to ensure the thermal response from the previous injection returned to baseline completely before the next injection.

1.3 Data Analysis

All ITC raw data (heat power-time curves) were processed using MicroCal Origin 7.0 software (OriginLab, USA) with the instrument. First, baseline correction was performed on the raw thermal signals to subtract the background thermal effect of buffer-buffer injection; then the corrected heat power was integrated to obtain the total enthalpy change (ΔH, unit: kJ/mol) for each injection, and a binding isotherm was plotted with "ligand/protein molar ratio (Mole Ratio)" as the abscissa and "integrated enthalpy change" as the ordinate.

A one-site binding model was used for non-linear fitting of the binding isotherm. During fitting, key thermodynamic parameters were calculated simultaneously: dissociation constant (KD, unit: μmol/L), binding enthalpy change (ΔH, unit: kJ/mol), binding entropy change (ΔS, unit: J/(mol·K),

derived from the formulas ΔG=ΔH-TΔS and ΔG=-RTln(1/KD), where T is absolute temperature and R is the gas constant), and number of binding sites (n). All experiments were independently repeated at least 3 times, and the results were expressed as "mean±SD".

2. Results and Analysis

2.1 Thermodynamic Characteristics of Binding Between Target Protein and Glucose

ITC measurements showed that glucose could specifically bind to the target protein at 25℃, with a typical binding curve shown in Figure 1 (corresponding to the glucose ITC curve mentioned earlier).

Heat power curve (upper curve): Obvious negative heat power peaks (exothermic reaction) were generated after each glucose injection. The peak area gradually decreased with increasing injection times and stabilized at the baseline level after the 18th injection, indicating that the protein binding sites were saturated.

Binding isotherm (lower curve): The integrated enthalpy change showed a trend of first linear increase and then plateau with the increase of "glucose/protein molar ratio", consistent with the one-site binding model. Non-linear fitting results showed that the binding parameters of glucose to the target protein were: dissociation constant KD=12.3±0.8 μmol/L, binding enthalpy change ΔH=-28.6±1.2 kJ/mol, number of binding sites n=0.98±0.03 (close to the theoretical value of 1.0), and binding entropy change ΔS=-35.2±2.1 J/(mol·K).

The above parameters indicate that the binding between glucose and the target protein has high affinity (KD at the 10^{-6} mol/L level), and the binding process is mainly enthalpy-driven (ΔH<0, ΔS<0), suggesting that hydrogen bonds or hydrophobic interactions are the main binding forces.

2.2 Thermodynamic Characteristics of Binding Between Target Protein and Mannitol

Compared with glucose, the binding behavior of mannitol to the target protein was significantly different, and its ITC binding curve is shown in Figure 2 (corresponding to the mannitol ITC curve mentioned earlier).

Heat power curve (upper curve): The intensity of negative heat power peaks generated after mannitol injection was significantly lower than that of glucose (peak area was about 1/5~1/4 of glucose), and more injections were required to reach saturation (stabilized at baseline after the 22nd injection), indicating that the binding between mannitol and the protein was weaker.

Binding isotherm (lower curve): The increasing trend of integrated enthalpy change was gentler, and the plateau enthalpy change value (-4.2±0.5 kJ/mol) was much lower than that of glucose. The fitted binding parameters were: KD=514.2±25.6 μmol/L, ΔH=-4.2±0.5 kJ/mol, n=1.02±0.04,

ΔS=12.8±1.8 J/(mol·K).

The data indicate that the binding affinity of mannitol to the target protein is significantly lower than that of glucose (KD value is about 42 times that of glucose), the contribution of enthalpy change to the binding process is greatly reduced, and the entropy change becomes positive, suggesting that the interaction at the binding interface between mannitol and the protein is weaker, possibly only forming a small number of hydrogen bonds or hydrophobic interactions.

2.3 Comparative Analysis of Binding Characteristics Between Glucose and Mannitol

Statistical comparison of the ITC parameters of the two ligands (Table 1) showed:

Affinity difference: The KD value of glucose (12.3 μmol/L) was significantly lower than that of mannitol (514.2 μmol/L), indicating that the target protein has higher specific recognition ability for glucose;

Thermodynamic driving difference: The binding of glucose is mainly enthalpy-driven (ΔH contributes 85.2% of ΔG), while the enthalpy change contribution of mannitol binding only accounts for 15.7% of ΔG, and entropy change becomes an important driving factor, suggesting that there are essential differences in the binding modes between the two and the protein;

Number of binding sites: The n values of both ligands are close to 1.0, indicating that there is only one specific binding site for hexitol ligands on the target protein, excluding the possibility of multi-site binding.

Identification of Downstream Target Genes

To identify the downstream target genes of the METTL3-IGF2BP3 axis, we integrated four datasets and established strict screening criteria for each, as follows:

1. MeRIP-seq data from METTL3-silenced cells

Purpose: To screen genes with METTL3-dependent m^6A modification.Screening criteria: m^6A peaks were identified using MACS2 software (q-value < 0.05) and mapped to the human genome; differential m^6A modifications were analyzed with DESeq2, with thresholds set as adjusted P-value (padj) < 0.05, fold change > 2, and each gene containing at least one significantly differential m^6A peak.

2. IGF2BP3-RIP-seq data

Purpose: To screen genes directly bound by IGF2BP3.Screening criteria: Binding peaks were

analyzed using HOMER software, and enrichment levels were calculated relative to IgG control; thresholds were set as enrichment fold > 2, enrichment significance P-value < 0.05, and binding peaks mainly localized to the 3'UTR region of genes.

3. Sh-IGF2BP3-RNA-seq data

Purpose: To screen genes regulated by IGF2BP3.Screening criteria: Differentially expressed genes were analyzed with DESeq2, with thresholds set as adjusted P-value (padj) < 0.05, fold change > 2, and gene expression level (FPKM) > 1.

4. HCC survival-associated genes

Purpose: To screen genes correlated with the prognosis of HCC patients.Screening criteria: The association between gene expression and overall survival (OS) was analyzed using the Cox proportional hazards regression model, with thresholds set as hazard ratio (HR) > 1 and prognosis association significance P-value < 0.05.

Overlap Analysis

Overlap analysis of the four datasets was sequentially performed using the R package "VennDiagram". Finally, genes that met all the aforementioned criteria were identified: HSPA5 and SLC39A10. Meanwhile, the regulatory effects of IGF2BP3 on HSPA5 and SLC39A10 were verified by PCR. The results showed that SLC39A10 was regulated by IGF2BP3, while HSPA5 exhibited no significant changes. Therefore, SLC39A10 was selected as the downstream target of IGF2BP3 for further investigation. These details and the intersection Venn diagram have been appropriately added to the main text and supplementary materials.

Statistical analysis

1. Experimental Reproducibility Design: All in vitro experiments were performed independently with at least 3 independent biological replicates to ensure the reliability and reproducibility of the results.

2. Statistical Software: Statistical analyses were conducted using SPSS version 24.0 (SPSS Inc., Chicago, IL, USA) or GraphPad Prism version 10.0 (GraphPad, CA, USA).

3. Data Presentation: All data are presented as mean ± SD.

4. Statistical Methods and Application Scenarios: Unpaired Student's t-test: Used for comparing differences between two independent sample groups (e.g., control group vs. single treatment group).

One-way analysis of variance (one-way ANOVA): Used for analyzing the overall differences among multiple sample groups under the influence of a single factor (e.g., 1 control group vs. 3 treatment groups with different concentrations). Two-way analysis of variance (two-way ANOVA): Used for analyzing the overall differences among multiple sample groups under the influence of two factors (e.g., different treatment times × different treatment concentrations). Chi-square test (χ^2 test): Used for comparing differences in categorical data (e.g., positive rate, incidence rate) between groups. Spearman's correlation analysis: Used for analyzing the correlation between non-normally distributed data or ordinal categorical data.

5. Post-hoc Tests for Multiple Group Comparisons: If one-way ANOVA or two-way ANOVA indicated significant differences among groups, post-hoc tests were further conducted to identify specific different groups: Pairwise comparisons among multiple groups in one-way ANOVA: Tukey's test was used; Pairwise comparisons among multiple groups in two-way ANOVA: Sidak's test was used; Comparisons between multiple groups and a single control group: Dunnett's test was used.

6. Verification of Statistical Method Assumptions: For t-tests and ANOVA, the following assumptions of the data were verified first: Normality: The Shapiro-Wilk test was used to verify whether the data conformed to a normal distribution; Homogeneity of variances: The Levene's test was used to verify whether the variances of the data in each group were homogeneous. All data used for t-tests or ANOVA in this study were confirmed to meet the above assumptions through the aforementioned tests; Spearman's correlation analysis does not rely on the assumption of normal distribution, so no relevant verification was performed.

7. Significance Criterion: A P-value < 0.05 was considered statistically significant.

Others

This study has several limitations that should be acknowledged. First, the STZ-induced diabetic mouse model used in this study mainly presents a type 1 diabetes-like phenotype caused by pancreatic β-cell destruction, which cannot fully recapitulate the pathological complexity of type 2 diabetes, the most prevalent metabolic comorbidity in patients with hepatocellular carcinoma (HCC). Thus, the findings of this study need to be further validated in more clinically relevant type 2 diabetic animal models in future work. Second, our current data revealed a close correlation between intracellular Zn^{2+}

homeostasis and immune cell infiltration in the HCC tumor microenvironment, while the direct causal relationship and underlying molecular mechanism have not been fully elucidated. Further in vitro immune cell co-culture assays and in vivo validation in immunocompetent mouse models are warranted to clarify the causal link between Zn^{2+} dysregulation and tumor immune microenvironment remodeling.

Supplementary tables

Table S1 Baseline data sheet

Parameters	Paraffin-embedded samples (n=80)	Fresh samples (n=16)
Age (year)	49 [38, 59]	50 [38, 58]
Sex		
Male	54 (67.5%)	11 (68.8%)
Female	26 (32.5%)	5 (31.2%)
TNM Stage		
I	17 (21.2%)	3 (18.8%)
II	31 (38.8%)	6 (37.5%)
III	26 (32.5%)	5 (31.2%)
IV	6 (7.5%)	2 (12.5%)

BCLC Stage		
0	21 (26.2%)	4 (25.0%)
A	28 (35.0%)	6 (37.5%)
B	16 (20.0%)	3 (18.8%)
C	15 (18.8%)	3 (18.8%)
D	0 (0.0%)	0 (0.0%)
Child-Pugh		
A	69 (86.2%)	14 (87.5%)
B	11 (13.8%)	2 (12.5%)
C	0 (0.0%)	0 (0.0%)
Differentiation		
Low	5 (6.2%)	1 (6.2%)
Middle	20 (25.0%)	4 (25.0%)
High	55 (68.8%)	11 (68.8%)
Combined hyperglycemic status		
Diabetes mellitus	28 (35.0%)	6 (37.5%)
Impaired glucose tolerance	16 (20.0%)	3 (18.8%)
Normal blood glucose	36 (45.0%)	7 (43.7%)
Fasting blood glucose		
Patients with hyperglycemia	8.2 [7.3, 9.5]	8.5 [7.5, 10.1]
Patients with normal glucose	5.4 [4.9, 5.8]	5.3 [4.8, 5.7]
Glycated hemoglobin		
Patients with hyperglycemia	7.3 [6.8, 8.1]	7.5 [7.0, 8.3]
Patients with normal glucose	5.6 [5.3, 5.8]	5.5 [5.2, 5.7]
Duration of hyperglycemia	5.2 [2.1, 8.5]	4.8 [1.8, 7.9]
Alcohol history	31 (38.8%)	5 (31.2%)
HBV infection	61 (76.2%)	10 (62.5%)
Liver cirrhosis	71 (88.8%)	12 (75.0%)

Table S2 RT-PCR primers

Primers	Sequences
h-METTL3 -F	TTGTCTCCAACCTTCCGTAGTGA
h-METTL3 -R	ATCAGAGAGGTGGTGTAGCAACTTC
h-IGF2BP3 -F	CAGATGCCAAACCAAAGACAG
h-IGF2BP3 -R	CTCACAGAGACAGGAGTTCAAAAGT
h-SLC39A10-F	CACCATCGTTTGCATCATCATC
h-SLC39A10-R	GCTCACTGGGAGTAATGGAATC
h-β-Actin-F	CATGTACGTTGCTATCCAGGC
h-β-Actin-R	CTCCTTAATGTCACGCACGAT
h-WTAP_qPCR_118bp_F1	ACATCCTTGTAATGCGACTAGCAA
h-WTAP_qPCR_118bp_R1	ACCATTGTTGATCTCAGTTGGG
h-METTL16_qPCR_118bp_F1	GGCGGAAGGCATAGTCGTT
h-METTL16_qPCR_118bp_R1	GTTTATGCTGGACCTTCAAATCAG
h-ALKBH5_qPCR_118bp_F1	CGCCGTCATCAACGACTACC
h-ALKBH5_qPCR_118bp_R1	CCCGAATAGGCTTGAACTGGA
h-METTL14_qPCR_118bp_F1	CTCTGTTCGTAAGCTCCCGGT
h-METTL14_qPCR_118bp_R1	GGCTTTTCACTATCCCGAGTACTT
h-METTL3_qPCR_118bp_F1	TTGTCTCCAACCTTCCGTAGTGA
h-METTL3_qPCR_118bp_R1	ATCAGAGAGGTGGTGTAGCAACTTC
h-FTO_qPCR_118bp_F1	TGGCGAAGGCGGCTTTAG
h-FTO_qPCR_118bp_R1	CTTAGCTTCGCGCTCTCGT
h-YTHDF1_qPCR_118bp_F1	TAACGACAACAAACCGGTCACA
h-YTHDF1_qPCR_118bp_R1	ATGGAGGTTGTGTGCTTGTAGG
h-YTHDF2_qPCR_118bp_F1	GACACATTCGCCTAGAGAACAAC
h-YTHDF2_qPCR_118bp_R1	TTTCAACACCTGCTTAGCCTTT
h-YTHDF3_qPCR_118bp_F1	TTTCTGGGTGTTTGCGAGC
h-YTHDF3_qPCR_118bp_R1	AGGATGCCAGGACACTGCA
h-YTHDC1_qPCR_118bp_F1	GTGCTAAAATGCTGGGAGGTGT
h-YTHDC1_qPCR_118bp_R1	TGGTGAGATGAGCCGACTTAGT
h-HNRNPC_qPCR_118bp_F1	TGTTGCATTGAGGAGTCAACTTG
h-HNRNPC_qPCR_118bp_R1	CTGGACCACAAAGCACTGATG
h-YTHDC2_qPCR_118bp_F1	GCAAGAAGAGAAACAACAAACCAC
h-YTHDC2_qPCR_118bp_R1	GCATCACCACCATCATCCAGTA
h-IGF2BP2_qPCR_118bp_F1	GAACATCACTAAGCAGACCCAGTC
h-IGF2BP2_qPCR_118bp_R1	CATGGATGGTGACAGGCTTCT
h-HNRNPG_qPCR_118bp_F1	AGATTACCCAAGTTCTCGTGATACT
h-HNRNPG_qPCR_118bp_R1	CGACCATATCCATCTCTATCGCT
h-ZC3H13_qPCR_118bp_F1	CTTCACCATCTTATCAGCGGAC
h-ZC3H13_qPCR_118bp_R1	GAGCGATGTCTTGGAGGACTC
h-HNRNPA2B1_qPCR_118bp_F1	GGTGCTCCTCGCAGAGTTGT
h-HNRNPA2B1_qPCR_118bp_R1	TCGCTTCAGCCCGATTTC
h-RBM15_qPCR_118bp_F1	AACAGCAACTTTCCTTCCAACA
h-RBM15_qPCR_118bp_R1	TTGAACCCTCCACAAGAAGACT
h-EIF3A_qPCR_118bp_F1	ACAGGCAGTGTTTGGACCTTC
h-EIF3A_qPCR_118bp_R1	CTTACGCGTGTATTGGAGGCA

h-EIF3D_qPCR_118bp_F1	CTTTGACCGCATCACCACGA
h-EIF3D_qPCR_118bp_R1	GGATGGCATCAGTGGCAAAC
h-SLC39A10(2941)_87bp_F1	CTATGCAGAAATAGAGATCGAACCA
h-SLC39A10(2941)_87bp_R1	CACACAAAAAATGTCTCCCCAAAG
h-SLC39A10(2941)_97bp_F1	CATGAACCAGTGTTGATATGTTTGA
h-SLC39A10(2941)_97bp_R1	CTGCATAGCCACAGAAGTTTCTACT

Table S3 CHIP primer

Primers	Sequences
hif1a-mettl3-chip-F1	TTCCCGAAATGTTTCCATTC
hif1a-mettl3-chip-R1	TCTAGCTGCCTCAGCCTACC
hif1a-mettl3-chip-F2	CACTCATGCAGGTAGCTCTCA
hif1a-mettl3-chip-R2	GGCATGCTTCTTTGTGTTGA
hif1a-mettl3-chip-F3	AGCACTTTGGGAGGCTGA
hif1a-mettl3-chip-R3	TGGTCTCAGCTCACTGCAAC
c-MYC-mettl3-chip-F1	TTCCCGAAATGTTTCCATTC
c-MYC-mettl3-chip-R1	TCTAGCTGCCTCAGCCTACC
c-MYC-mettl3-chip-F2	TCTGTACTATGGCCGGGTGT
c-MYC-mettl3-chip-R2	CTCCCGGGTTCAAGTGATTA
c-MYC-mettl3-chip-F3	AGCACTTTGGGAGGCTGA
c-MYC-mettl3-chip-R3	TGGTCTCAGCTCACTGCAAC

Table S4 Antibody information

Antibody Name	Merchant	Item Number
m6A Ab	abcam	ab208577
p-AKT Ab	abcam	ab192623
AKT Ab	abcam	ab214166
p-AMPKα Ab	Immunoway	PT1374R
AMPKα Ab	Immunoway	PT0165R
p-EGFR Ab	Proteintech	84906-1-RR
EGFR Ab	Proteintech	18986-1-AP
ADAM17 Ab	Proteintech	84292-4-RR
METTL3 Ab	Proteintech	15073-1-AP
IGF2BP3 Ab	Proteintech	14642-1-AP
c-MYC Ab	Proteintech	10828-1-AP
SLC39A10 Ab	Immunoway	YN1549
FLAG Tag Ab	Proteintech	66008-4-Ig
β-Actin Ab	Proteintech	66009-1-Ig
Normal Rabbit IgG	Proteintech	SA00001-2
Normal Mouse IgG	Proteintech	SA00001-1

Supplementary figures

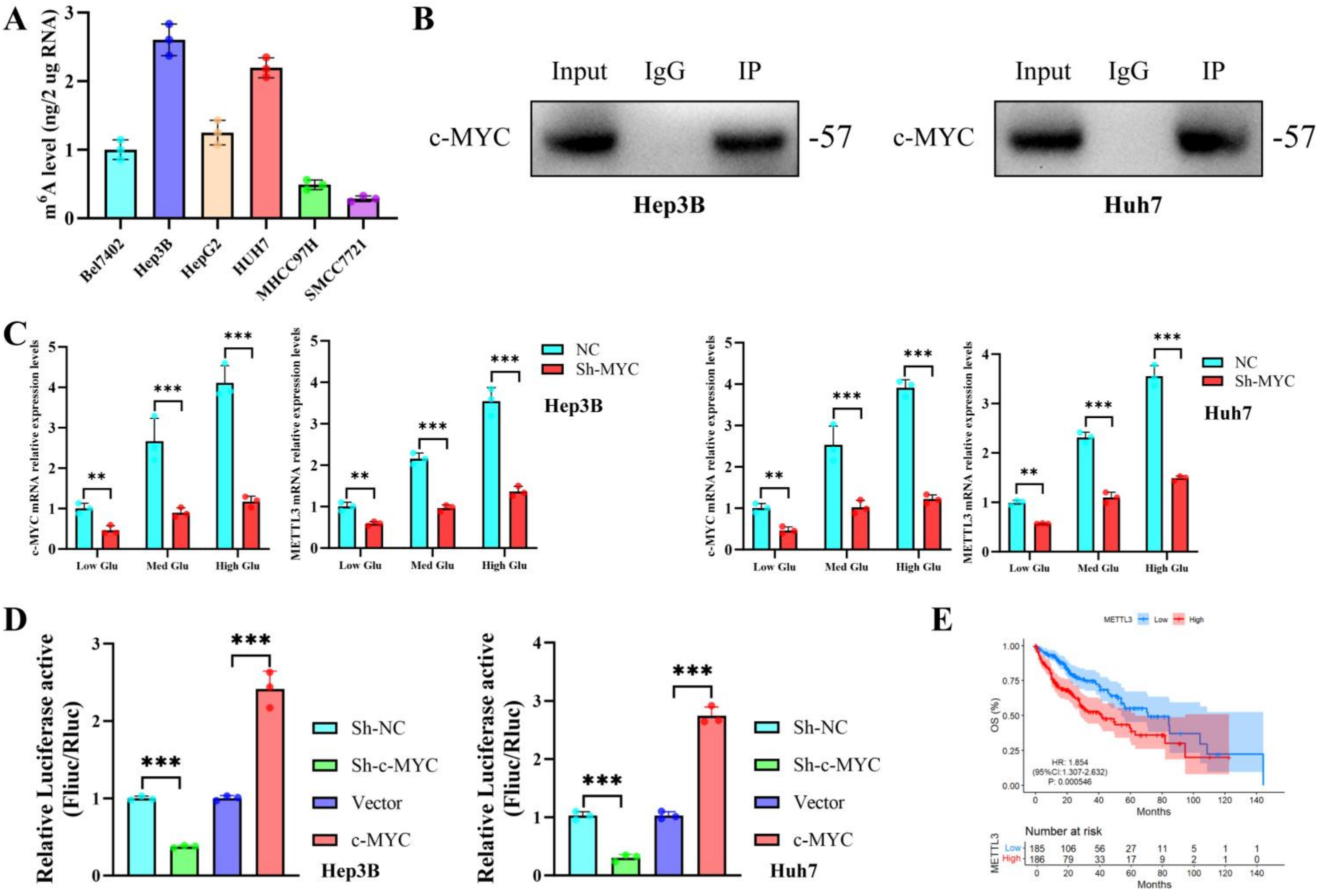

Figure S1 Glucose Metabolism Promotes METTL3 Expression via c-MYC in Hepatocellular Carcinoma

(A) Among six common hepatocellular carcinoma cell lines, Hep3B and HUH7 displayed the highest m6A methylation levels; (B) c-MYC antibody validation; (C) The expression of c-MYC was regulated by glucose concentrations; (D) Dual-luciferase reporter assays verified that c-MYC could directly bind to the METTL3 promoter and thereby modulate its transcription; (E) METTL3 expression was significantly correlated with the prognosis of hepatocellular carcinoma in the TCGA-LIHC cohort.

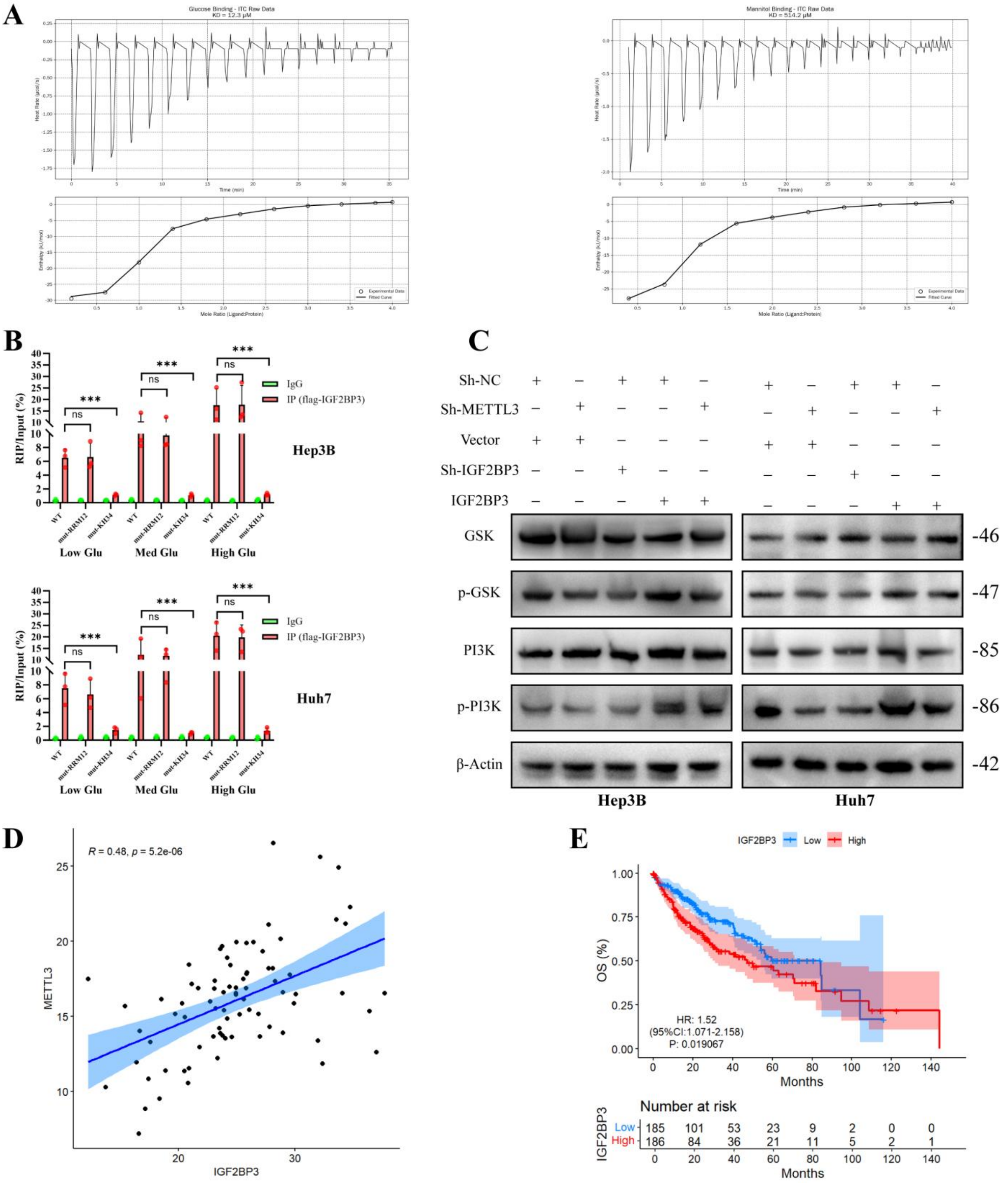

Figure S2 IGF2BP3 Binds Directly to Glucose

(A) Isothermal Titration Calorimetry (ITC) assays demonstrated that IGF2BP3 could directly bind to glucose with a KD value of 12.3 μmol/L, but failed to directly bind to the glucose analog mannitol (KD value: 514.2 μmol/L); (B) IGF2BP3 bound to and regulated the m^{6}A methylation of SLC39A10 mRNA via its KH34 domain; (C) Both METTL3 and IGF2BP3 could activate the AKT pathway, and knockdown of METTL3 reversed the IGF2BP3-induced activation of the AKT pathway; (D) METTL3 and IGF2BP3 exhibited a strong correlation at the RNA level (R=0.48,<0.001);(E)

IGF2BP3 expression was significantly correlated with the prognosis of hepatocellular carcinoma in the TCGA-LIHC cohort.

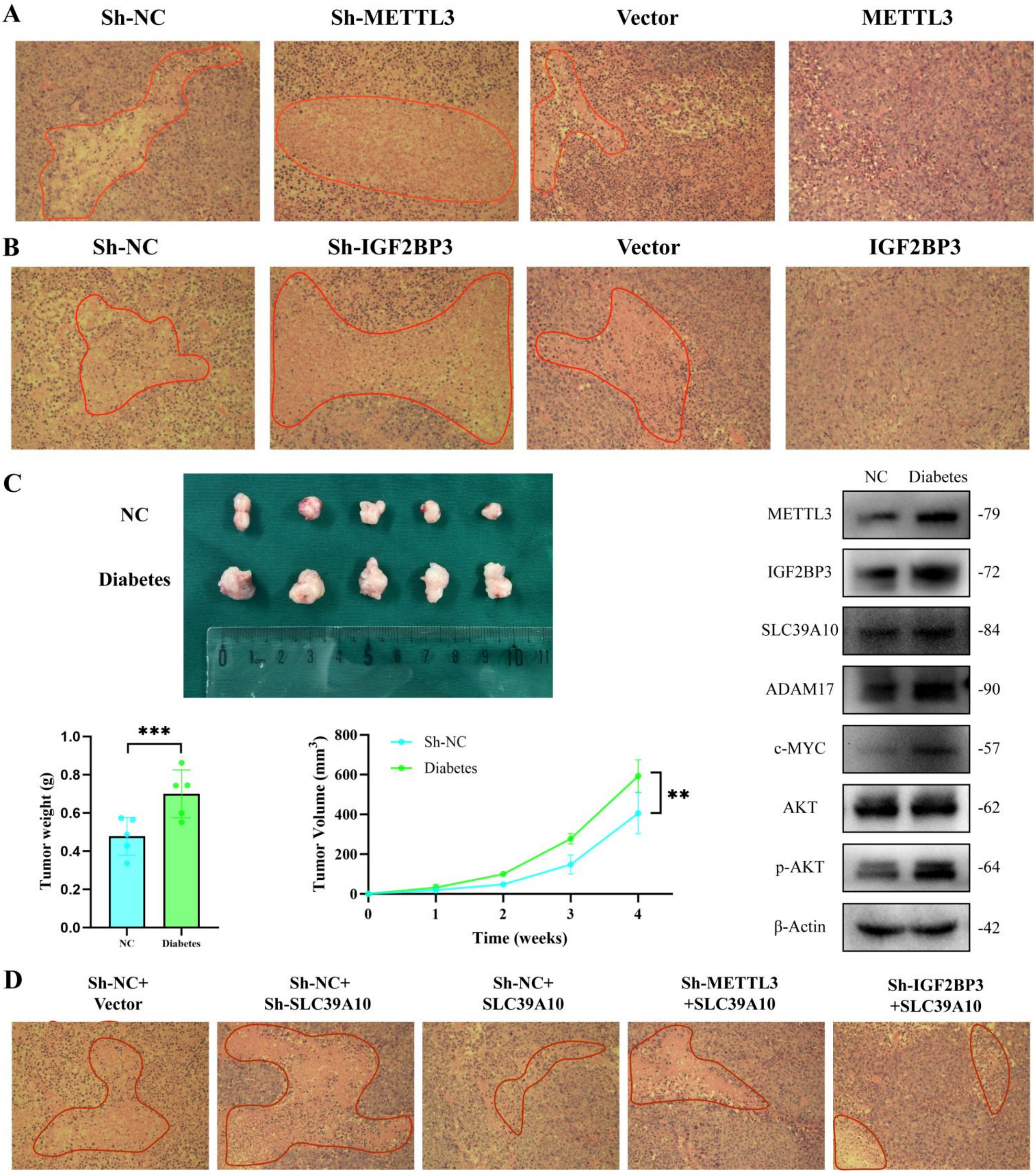

Figure S3 METTL3 and IGF2BP3 Promote Tumor Progression

(A-B) The subcutaneous tumors in the METTL3 and IGF2BP3 high-expression groups exhibited a higher necrosis ratio, while those in the low-expression groups showed a lower necrosis ratio; (C)

Compared with the control group, diabetic mice exhibited larger subcutaneous tumors with faster growth rates, and higher expression levels of METTL3, IGF2BP3, SLC39A10, ADAM17, c-MYC, and p-AKT in tumor tissues; (D) The subcutaneous tumors in the SLC39A10 overexpression group had a higher necrosis ratio, and this phenomenon could be reversed by knockdown of METTL3 or IGF2BP3.

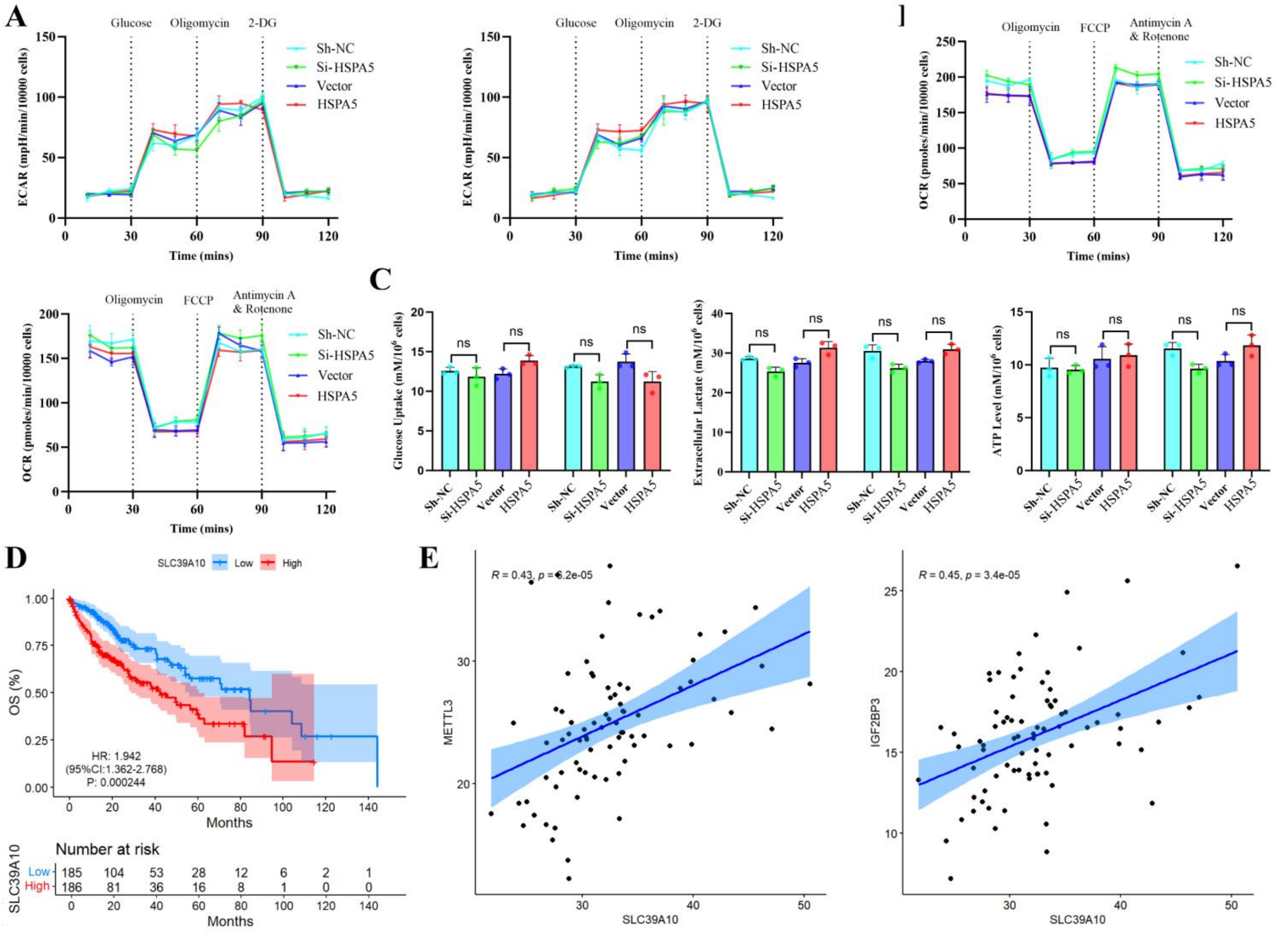

Figure S4 METTL3 and IGF2BP3 Promote SLC39A10 Expression

(A-C) HSPA5 had no significant effect on glycolysis in hepatocellular carcinoma cells; (D) SLC39A10 expression was significantly correlated with the prognosis of hepatocellular carcinoma in the TCGA-LIHC cohort; (E) METTL3/IGF2BP3 was significantly correlated with SLC39A10 at the RNA level.

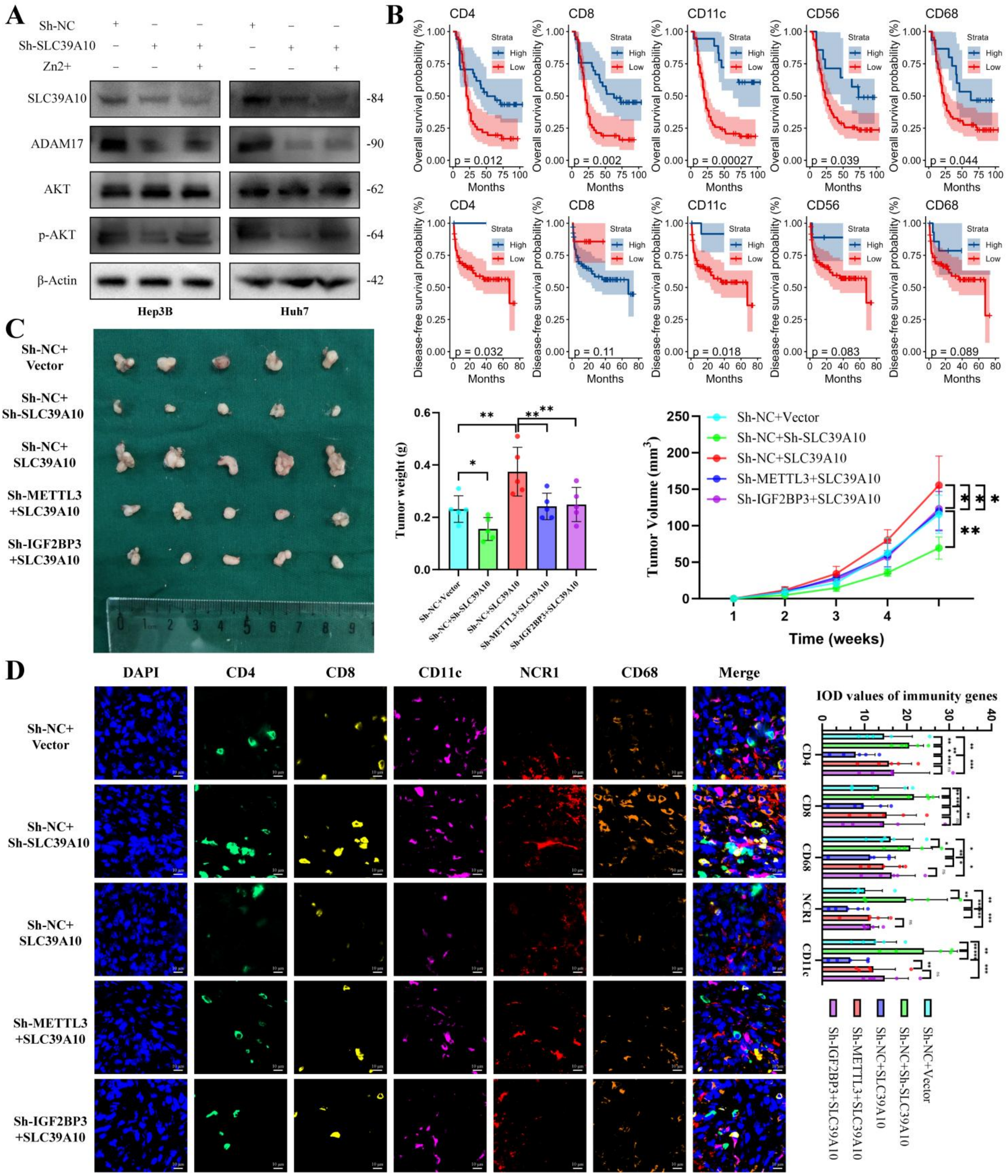

Figure S5 METTL3 and IGF2BP3 Regulate Hepatocellular Carcinoma Progression via SLC39A10

(A) Knockdown of SLC39A10 inhibited the ADAM17 and AKT signaling pathways; exogenous zinc ion supplementation reversed the inhibition of the AKT pathway, but failed to restore ADAM17 expression; (B) CD4, CD8, CD11c, CD56, and CD68 were all associated with overall survival (OS) in hepatocellular carcinoma (HCC), whereas only CD4 and CD11c correlated with disease-free survival

(DFS); (C) In C57BL/6J mice, SLC39A10 promoted tumor proliferation and tumorigenesis, and this effect could be reversed by knockdown of METTL3 or IGF2BP3; (D) In C57BL/6J mice, the proportions of CD4, CD8, CD11c, NCR1, and CD68 were lower in the SLC39A10 overexpression group, and this phenomenon was reversed by knockdown of METTL3 or IGF2BP3.

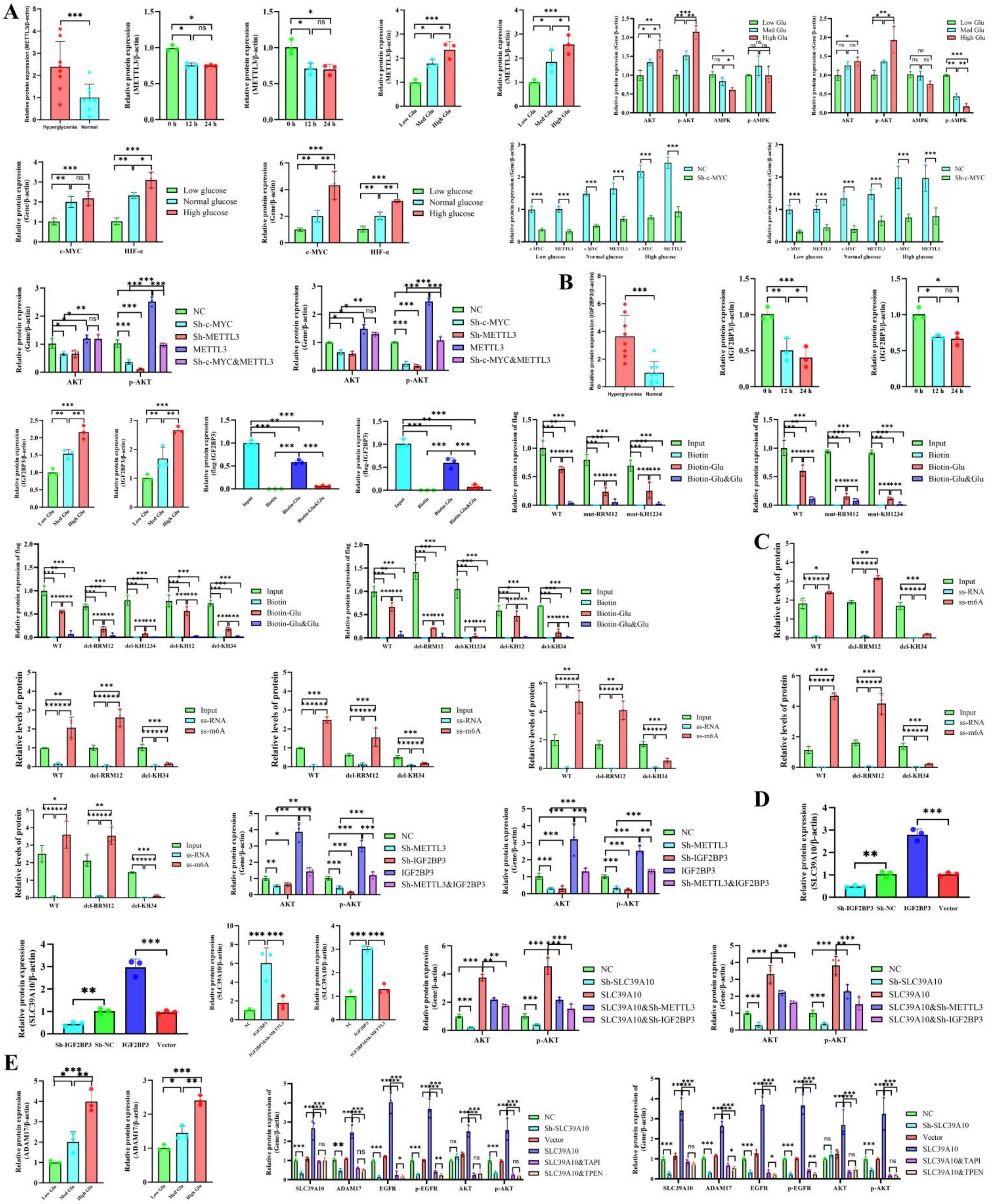

Figure S6 Quantitative Analysis of Western Blot Data

(A) Quantitative presentation of all WB data in Figure 1; (B) Quantitative presentation of all WB data in Figure 2;(C) Quantitative presentation of all WB data in Figure 3; (D) Quantitative presentation of all WB data in Figure 4; (E) Quantitative presentation of all WB data in Figure 5.
